# Innervation of adipocytes is limited in mouse perivascular adipose tissue

**DOI:** 10.1152/ajpheart.00041.2024

**Published:** 2024-05-24

**Authors:** Marie Hanscom, Wilmarie Morales-Soto, Stephanie W. Watts, William F. Jackson, Brian D. Gulbransen

**Affiliations:** ^1^Department of Physiology, Michigan State University, East Lansing, Michigan, United States; ^2^Department of Physiology and Biomedical Engineering, Mayo Clinic, Rochester, Minnesota, United States; ^3^Department of Pharmacology and Toxicology, Michigan State University, East Lansing, Michigan, United States

**Keywords:** adipocytes, innervation, perivascular adipose tissue

## Abstract

Perivascular adipose tissue (PVAT) regulates vascular tone by releasing anticontractile factors. These anticontractile factors are driven by processes downstream of adipocyte stimulation by norepinephrine; however, whether norepinephrine originates from neural innervation or other sources is unknown. The goal of this study was to test the hypothesis that neurons innervating PVAT provide the adrenergic drive to stimulate adipocytes in aortic and mesenteric perivascular adipose tissue (aPVAT and mPVAT), and white adipose tissue (WAT). Healthy male and female mice (8–13 wk) were used in all experiments. Expression of genes associated with synaptic transmission were quantified by qPCR and adipocyte activity in response to neurotransmitters and neuron depolarization was assessed in *Adipoq^Cre+^;GCaMP5g-tdT^f/WT^* mice. Immunostaining, tissue clearing, and transgenic reporter lines were used to assess anatomical relationships between nerves and adipocytes. Although synaptic transmission component genes are expressed in adipose tissues (aPVAT, mPVAT, and WAT), strong nerve stimulation with electrical field stimulation does not significantly trigger calcium responses in adipocytes. However, norepinephrine consistently elicits strong calcium responses in adipocytes from all adipose tissues studied. Bethanechol induces minimal adipocyte responses. Imaging neural innervation using various techniques reveals that nerve fibers primarily run alongside blood vessels and rarely branch into the adipose tissue. Although nerve fibers are associated with blood vessels in adipose tissue, they demonstrate limited anatomical and functional interactions with adjacent adipocytes, challenging the concept of classical innervation. These findings dispute the significant involvement of neural input in regulating PVAT adipocyte function and emphasize alternative mechanisms governing adrenergic-driven anticontractile functions of PVAT.

**NEW & NOTEWORTHY** This study challenges prevailing views on neural innervation in perivascular adipose tissue (PVAT) and its role in adrenergic-driven anticontractile effects on vasculature. Contrary to existing paradigms, limited anatomical and functional connections were found between PVAT nerve fibers and adipocytes, underscoring the importance of exploring alternative mechanistic pathways. Understanding the mechanisms involved in PVAT’s anticontractile effects is critical for developing potential therapeutic interventions against dysregulated vascular tone, hypertension, and cardiovascular disease.

## INTRODUCTION

Hypertension is the leading contributor to global disease burden and affects nearly 1.3 billion individuals worldwide (1). Chronic high blood pressure damages multiple organ systems and often goes undiagnosed because of the absence of symptomology in ∼46% of adults (2–9). Several risk factors such as genetics and lifestyle contribute to the development and progression of hypertension; yet many of the mechanisms contributing to the onset and pathoprogression of hypertension remain uncharacterized. Perivascular adipose tissue (PVAT) has emerged as a key regulator of vascular tone and blood pressure (10–14).

PVAT is the specialized collection of adipose tissue that surrounds the majority of the body’s vasculature, excluding capillaries, pulmonary, and cerebral blood vessels. In addition to adipocytes, PVAT also contains immune cells, antigen presenting cells, fibroblasts, nerve fibers, microvasculature, and extracellular matrix proteins. Unique phenotypes of PVAT are present in differing anatomical locations and are the result of heterogeneity in adipocyte progenitors, differentiation, cellular morphology and composition, as well as species-specific differences (14–26). PVAT was originally proposed to serve a static, structural role; however, it is now recognized as an active player in vascular functions and exerts regulatory effects on vascular tone that are lost in disease. Soltis and Cassis (27) provided the first evidence of vasoactive effects of PVAT in a seminal study showing that PVAT surrounding the rat thoracic aorta diminished the contractile response induced by noradrenaline. These observations have since been replicated in multiple species and PVAT regions, all of which support the suggestion that the presence of PVAT exerts an anticontractile effect on blood vessels that decreases blood pressure (28–30). Notable exceptions are found in pigs where PVAT has a procontractile effect on coronary arteries (19, 31) and rats, wherein uterine PVAT regulates uterine artery tone and blood flow during pregnancy by exerting procontractile effects in the absence of pregnancy and antidilatory effects during pregnancy on uterine arteries (32–34).

Vasoactive actions of perivascular adipocytes are mediated by several bioactive factors including adipokines, cytokines, hormones, and vasoactive substances (25), which induce vasodilation through both endothelium-dependent and -independent pathways (25, 30, 35–41). Their release from PVAT, and subsequent vasodilatory effects, can be driven by neurotransmitters including norepinephrine, which paradoxically functions as a sympathetic catecholamine and vasoconstrictor. Norepinephrine primarily acts on β_3_-adrenergic receptors expressed by adipocytes, generating nitric oxide and adiponectin, among other factors, which then, in turn, stimulate vasodilation (35, 42). This mechanism is postulated to provide negative feedback to counteract vasoconstriction induced by adrenergic signaling.

Despite the central role of norepinephrine in driving the anticontractile effects of PVAT, the relevant sources of norepinephrine remain enigmatic. The prominence of norepinephrine in sympathetic neurotransmission has led to widespread speculation that PVAT is controlled by innervation from sympathetic neurons. This concept is supported by data showing sympathetic arborizations in white adipose tissue (WAT) and functional effects of norepinephrine on inguinal and mesenteric adipocytes (35, 42, 43). Sympathetic and sensory nerves are present within adipose tissue, but their extent varies and their functional relationship with adipocytes is unclear (42–54). Suppression of neurotransmission with tetrodotoxin has yielded conflicting results regarding functional neural innervation of adipose tissue with findings both supporting and negating neuroregulation of PVAT anticontractile responses. Tetrodotoxin treatment either mitigates PVAT anticontractile effects following nerve depolarization with electrical field stimulation or has no effect (42, 55). Conclusive evidence of direct, classical neural communication with perivascular adipocytes, with norepinephrine as the intermediary, has yet to be established.

Our goal in this study was to provide a resolution to the question of potential neural control and innervation of PVAT by directly testing the hypothesis that adipocytes in PVAT are innervated by nerves. To this end, we used a combination of contemporary anatomical labeling and imaging techniques alongside functional calcium imaging studies of neurotransmission in PVAT. The data indicate a surprisingly limited potential for direct neuron-adipocyte communication in multiple adipose tissue depots. These observations raise the possibility that adrenergic control of PVAT anticontractile functions is independent of neural control and results from nonneuronal mechanisms.

## MATERIALS AND METHODS

### Animals

Male and female mice, 8–13 wk in age, were included in this study. All female mice were virgin mice with no history of mating. Mice expressing the genetically encoded Ca^2+^ indicator (GECI), GCaMP5g, with tdTomato reporter protein (tdT), in adipocytes were generated by breeding B6;*129S6-Polr2a^Tn(pb-CAG-GCaMP5g,-tdTomato)Tvrd^/*J mice (Jackson Laboratory, Stock No. 024477; RRID: IMSR_JAX:024477) with B6.*FVB-Tg(Adipoq-cre)1Evdr*/J mice (Jackson Laboratory, Stock No. 028020; RRID: IMSR_JAX:028020). Offspring, herein referred to as *Adipoq^Cre^;GCaMP5g-tdT* mice, were used for ex vivo calcium imaging and immunofluorescence studies. Additional transgenic mouse lines were included to confirm immunofluorescence findings; *NPY-GFP*, *TRPV1^Cre^;GCaMP5g-tdT*, *Baf53^Cre^;GCaMP5g-tdT*, *Wnt1^Cre^;GCaMP5g-tdT,* and *Thy1-GFP*. Homozygous *NPY-GFP* mice, B6.FVB-Tg*^(Npy-hrGFP)1Lowl^*/J, were obtained from Jackson Laboratory (Stock No. 006417; RRID: IMSR_JAX:006417). *TRPV1^Cre^;GCaMP5g-tdT* mice expressing GCaMP5g and tdT under control of the TRPV1 promoter were generated by breeding B6;*129S6-Polr2a^Tn(pb-CAG-GCaMP5g,-tdTomato)Tvrd^/*J mice (Jackson Laboratory, Stock No. 024477; RRID: IMSR_JAX:024477) with TRPV1^cre^ mice (B6.129-Trpv1tm1^(cre)Bbm/J^, The Jackson Laboratory, Stock No. 017769, RRID: IMSR_JAX:017769). *Baf53^Cre^;GCaMP5g-tdT* mice expressing GCaMP5g and tdT under control of the Baf53 promoter were generated by breeding B6;*129S6-Polr2a^Tn(pb-CAG-GCaMP5g,-tdTomato)Tvrd^/*J mice (Jackson Laboratory, Stock No. 024477; RRID: IMSR_JAX:024477) with BAF53b^Cre^ mice (Jackson Laboratory, Tg(Actl6b-Cre)*^4092Jiwu/J^*, Stock No. 027826; RRID: IMSR_JAX:027826). *Wnt1^Cre^;GCaMP5g-tdT* mice, expressing GCaMP5g and tdT under control of the neural-crest promoter, Wnt1, were generated by breeding B6;*129S6-Polr2a^Tn(pb-CAG-GCaMP5g,-tdTomato)Tvrd^/*J mice with B6.Cg-E2f1^Tg(Wnt1-cre)2Sor^/J mice (Jackson Laboratory, Stock No. 022501; RRID: IMSR_JAX:022501). Male and female *Thy1-GFP* mice were obtained from Jackson Laboratory (Tg(Thy1-EGFP)MJrs/J, Stock No. 007788, RRID: IMSR_JAX:007788). *Wnt1^Cre^;GCaMP5g-tdT* and *Adipoq^Cre^;RC::L-hM3Dq* transgenic mice, were included as positive controls for ex vivo calcium imaging studies. *Adipoq^Cre^;RC::L-hM3Dq* mice were generated by breeding B6;*129S6-Polr2a^Tn(pb-CAG-GCaMP5g,-tdTomato)Tvrd^/*J mice with B6.Cg-Gt(ROSA)26Sor^tm3.3(CAG-EGFP,-CHRM^*^/mCherry/Htr2a)Pjen^/J mice (Jackson Laboratory, Stock No. 026943, RRID: IMSR_JAX:026943). *Adipoq^Cre−^;RC::L-hM3Dq^f/WT^* mice have constitutive expression of enhanced green fluorescent protein (EGFP) in all cells in the absence of Cre allowing for visualization of vasculature in adipose tissue. All mouse genotypes were verified commercially by Transnetyx (Boston, MA).

Mice were housed in Optimice Cages (Animal Care Systems, Centennial, CO) with a half-inch corncob bedding (Frontier Distributing, Oxford, MI), cotton and paper nestlets (NES3600, Ancare, Bellmore, NY), and red mouse igloos (K3327, Bio-Serve, Flemington, NJ) for environmental enrichment. Mice had free access to chow (Teklad Irradiated Global 19% Protein Extruded Rodent Diet, 2919, Inotiv, Lafayette, IN) and water (tap distilled) and were maintained on 12-h:12-h light/dark cycles, under ambient environmental conditions of 19–24°C and 40–60% humidity. All animal experiments were approved by the Michigan State University Institutional Animal Care and Use Committee and conducted in accordance with the standards established by the National Institutes of Health (NIH) Guide for the Care and Use of Laboratory Animals in facilities accredited by the Association for Assessment and Accreditation for Laboratory Care (AAALAC) (1) (AUF No. PROTO202000009).

### Drugs and Reagents

Bethanechol chloride (1071009, Sigma, St. Louis, MO) and (±)-norepinephrine (+)-bitartrate salt (NE; A7256, Sigma) were prepared according to the manufacturer’s instructions and used at a final concentration of 10 mM in 1× Kreb’s buffer. Both bethanechol and NE were applied to adipose tissue by bath application using gravity perfusion. DMEM-F12 (11039021, Thermo Fisher Scientific, Pittsburg, PA) and 1× Kreb’s buffer were used in calcium imaging experiments. Kreb’s buffer contained (in mM) 121 NaCl (Sigma, 746398), 5.9 KCl (Sigma, 74636), 2.5 CaCl_2_ (Sigma, C1016, 500 g), 1.2 MgCl_2_ (Thermo Fisher Scientific, 3818), 1.2 NaH_2_PO_4_ (Thermo Fisher Scientific, 2444-01), 10 HEPES (Sigma, H3375), 21.2 NaHCO_3_ (Thermo Fisher Scientific, S233), 1 Na pyruvate (Sigma, P5280), and 8 mM glucose (Sigma, G7021). Phosphate-buffered saline used in immunofluorescence studies contained 137 mM NaCl, 2.7 M KCl, 10 M Na_2_HPO_4_ (Thermo Fisher Scientific, 3828-01), 1.8 mM KH_2_PO_4_ (Thermo Fisher Scientific, 3246-01), and 0.02% Na azide (Sigma, S2002). Ultrapure water was purchased from Thermo Fisher Scientific (SH30538.03).

### Antibodies and Dyes

Antibodies and dyes used in this study include anti-rat-CD31 (15 µL antibody in 100 µL sterile normal saline via tail vein injection, Abcam, ab56299, RRID:AB_940884, Waltham, MA), peripherin (A-3) (1:100 dilution, Santa Cruz Biotechnology, sc-377093, RRID:AB_2923264, Santa Cruz, CA), anti-rat tyrosine hydroxylase (1:200 dilution, AbD Serotec, AHP1879, RRID:AB_2201649, Hercules, CA), anti-P92 (advillin) (1:100 dilution, Abcam, ab72210, RRID:AB_1951510), PGP9.5 (1:100 dilution, Neuromics, GP14104, RRID:AB_2210625, Edina, MN), PGP9.5175-191 (1:100 dilution, Neuromics, RA12103, RRID:AB_2315126), anti-β-tubulin 3 (TUJ) (1:100 dilution, Aves Labs, TUJ, RRID:AB_2313564, Davis, CA), NeuO (100 µL ip injection, 01801, Stem Cell Technologies, Cambridge, MA), lectin 649 (100 µL via tail vein injection and 75 µL via transcardial perfusion, DL-1178-1, Vector, Newark, CA), and l-noradrenaline (norepinephrine; 1:100 dilution, Immusmol, IS1042, Bordeaux, France). Secondary antibodies include donkey anti-rat IgG Alexa Fluor 488 (1:500 dilution, JacksonImmuno Research, 712-545-150, RRID:AB_2340683, West Grove, PA), donkey anti-mouse IgG Alexa Fluor 594 (1:500 dilution, JacksonImmuno Research,715-585-150, RRID:AB_2340846), donkey anti-chicken IgG Alexa Fluor 488 (1:500 dilution, JacksonImmuno Research, 703-545-155, RRID:AB_2340375, RRID:AB_2340375), donkey anti-guinea pig IgG Alexa Fluor 488 (1:500 dilution, JacksonImmuno Research, 706-545-148, RRID:AB_2340472), donkey anti-rabbit IgG Alexa Fluor 594 (1:500 dilution, JacksonImmuno Research, 711-585-152, RRID:AB_2340621), donkey anti-sheep IgG Alexa Fluor 488(1:500 dilution, JacksonImmuno Research, 713-545-003, RRID:AB_2340744), and donkey anti-rat IgG Alexa Fluor 594 (1:500 dilution, JacksonImmuno Research, 712-585-153, RRID:AB_2340689).

### In Vivo Vasculature Labeling

In vivo labeling of the mouse vasculature was performed by tail vein injection with anti-CD31 (15 µL antibody in 100 µL sterile normal saline, Abcam, ab56299, RRID:AB_940884) and/or lectin-649 (100 µL, DL-1178-1, Vector) as previously described (51, 56). Mice were briefly anesthetized with 3% isoflurane supplied in 100% O_2_ for 2 min in an induction chamber and then placed in a restraint tube to expose the tail while limiting animal movement. The tail was wiped with a sterile ethanol pad and the lateral tail vein was identified with the aid of an illuminator (Veinlite-R, Translite, Sugarland, TX). Anti-CD31 antibody (15 µL antibody in 100 µL normal sterile saline; Abcam, ab56299, RRID:AB_940884) was injected into the lateral tail vein using a 31-g insulin needle (0.31 × 8 mm, 0.5 mL, U-100, Thermo Fisher Scientific). The mouse was removed from the restraint tube and returned to its home cage for 30 min to allow for full circulation of the antibody (56). After 30 min, mice injected with CD31 were taken for adipose tissue collection as indicated in *Tissue Collection and Preparation*. For lectin labeling, 100 mL of lectin (DL-1178-1, Vector) was injected into the lateral vein using a 31-g insulin needle. After 15 min, an additional 75 mL of lectin was administered transcardially, and allowed to circulate for 3 min, before saline perfusion and tissue collection, as indicated in *Tissue Collection and Preparation*.

### Tissue Collection and Preparation

#### For ex vivo calcium imaging.

**Figure 1.**
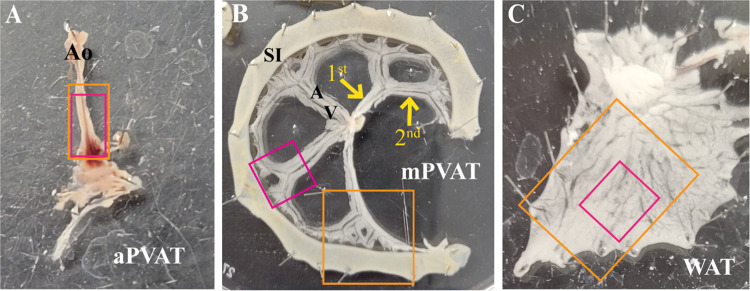
Tissue dissection for molecular and imaging studies. Schematic of adipose tissue dissection for ex vivo live calcium imaging and immunofluorescence in the aortic perivascular adipose tissue (aPVAT; *A*), mesenteric perivascular adipose tissue (mPVAT; *B*), and white adipose tissue (WAT; *C*). Orange boxes are representative of dissected tissue taken for immunofluorescent staining. Magenta boxes are representative of tissue dissected for ex vivo live calcium imaging. Primary and secondary branching of the vasculature in the mPVAT is indicated by yellow arrows (*B*). Ao, aorta; A, artery; SI, small intestines; V, vein, 1st, primary branching; 2nd, secondary branching.

Aortic and mesenteric PVAT (aPVAT, mPVAT) and white adipose tissue (WAT) were collected from male and female *Adipoq^Cre+^;GCaMP5g-tdT^fl/WT^* mice. Animals were anesthetized by induction with 3% isoflurane supplied in 100% O_2_ for 3 min in an induction chamber. After mice had reached a surgical plane of anesthesia with no observed reflex responses to hind paw pinch, the mice were subjected to cervical dislocation. The mice were secured in a supine position, the abdominal cavity opened to access mPVAT and perigonadal white adipose tissue (WAT), and the rib cage was opened to access aPVAT. The left and right WAT lateral to the bladder were isolated and placed in ice-cold DMEM-F12 (11039021, Thermo Fisher Scientific). The mPVAT was isolated by dissecting the ileum from the cecum and the duodenum from the stomach and gently removing the small intestines with the mesentery intact to ice-cold DMEM-F12. Thoracic aPVAT was isolated by removing the lungs and dissecting the aorta with PVAT intact from above the diaphragm to the aortic arch, placing in ice-cold DMEM-F12. All tissue was kept on ice in DMEM-F12 until ready for further dissection for calcium imaging. For calcium imaging preparation, the adipose tissue was pinned out, with gentle stretching, using insect pins (26002-20, Thermo Fisher Scientific) in sylgard agar coated dishes (50-366-794, Thermo Fisher Scientific) containing ice-cold DMEM-F12. Small sections of the mPVAT (containing two “Y” shaped secondary vascular branches connecting to the small intestines and the primary vascular branch), sections of the aPVAT (∼5 mm × 3 mm), and WAT (∼5 mm) were cut (Fig. 1) and pinned into sylgard agar coated custom in-house three-dimensional (3-D)-printed rectangular imaging dishes (dish, 8 cm × 5.6 cm; imaging chamber, 2.1 cm × 1.5 cm) containing ice-cold DMEM-F12 and placed, covered, on ice until ready to use in live-cell calcium imaging. The prepared adipose tissue was incubated at 37°C in 5% CO_2_ for 30 min before calcium imaging. mPVAT was collected from *Adipoq^Cre−^;RC::L-hM3D^f/−^* in a similar manner as described earlier, with the additional step of gently clearing the adipocytes from the top, but not the remaining surfaces, of the mesenteric artery to allow for clear visualization of the mesenteric artery (*n* = 3 females).

For collection of colonic tissue from a single *Wnt1^Cre+^;GCaMP5g-tdT^f/−^* mouse, the colon was isolated, from the cecum to rectum, and placed into ice-cold DMEM-F12 until ready for preparation. Full-thickness colon was opened along the mesenteric border and pinned out, stretched flat with the mucosa up, in a sylgard agar dish, using insect pins in ice-cold DMEM-F12. Whole mount circular muscle myenteric plexus (CMMP) preparations were obtained by dissection of the mucosa at the level of the lamina propria and longitudinal muscle and serosa from the circular muscular layer to expose the CMMP. Small sections of the distal CMMP preparation (∼7 mm × 5 mm) were then pinned into imaging dishes and kept, covered, on ice until ready to use in live-cell calcium imaging. The prepared colon pieces were incubated at 37°C in 5% CO_2_ for 30 min before calcium imaging (57).

#### For molecular studies.

mPVAT, aPVAT, and WAT were collected from three male and three female *Adipoq^Cre−^;GCaMP5g-tdT^fl/WT^* mice. mPVAT and aPVAT were isolated and dissected as described earlier, adipocytes were further dissected away from the large vasculature, then placed into 1.5-mL microcentrifuge tubes, and flash frozen in liquid nitrogen. WAT was removed from the animals, directly placed into 1.5-mL sterile microcentrifuge tubes, and flash frozen in liquid nitrogen. One brain was collected to serve as a positive control in qPCR studies (*n* = 1 female). After removal of the adipose tissue, the brain was removed from the mouse skull using grangers, placed on to an ice-cold cutting mat. The left and right cortex and hippocampus were dissected, placed into separate 1.5-mL sterile microcentrifuge tubes, and flash frozen in liquid nitrogen. Frozen tissue was stored at −80°C.

#### For histological analyses.

For mice injected with anti-CD31 antibody, adipose tissue was collected as described earlier for calcium imaging studies, leaving the intestines attached in the mPVAT, and fixed for 2 h at room temperature in the dark with 4% paraformaldehyde (PFA, 158127, Sigma), followed by 3 × 10-min washes to remove the PFA, and stored in 1× PBS in the dark at 4°C. For mice injected with lectin, animals were anesthetized by induction with 3% isoflurane supplied in 100% O_2_ for 3 min in an induction chamber. After mice had reached a surgical plane of anesthesia with no observed reflex responses to hind paw pinch, the mice were removed to a nose cone supplied with 1.5% isoflurane-100% O_2_. Absence of reflex was confirmed, and mice were secured in a prone position, the abdominal cavity opened, a sterile gauze pad soaked in prewarmed, sterile 0.9% saline was (746398, Sigma) placed over the exposed organs to retain moisture, and 75 µL of lectin was transcardially injected without opening the diaphragm using a 31-g insulin needle. The needle was held in place for 1 min following injection, and after 3 min, the mouse was perfused with 5 mL of ice-cold sterile 0.9% saline. Total time under 1.5% isoflurane-100% O_2_ was 7–9 min. The aPVAT, mPVAT, and WAT were collected as described earlier for calcium imaging studies. The tissue was fixed for 2 h at room temperature in the dark with 4% PFA, followed by three 10-min washes to remove the PFA, and stored in 1× PBS in the dark at 4°C.

### RNA Extraction and cDNA Synthesis

RNA was extracted and purified from frozen adipose and brain tissue using QIAzol reagent and the miRNeasy Mini Kit according to the manufacturer’s instructions (QGN-217004, Qiagen, Hilden, Germany). Cells were lysed with the aid of mechanical dissociation (Bead Bug, Stellar Scientific, Baltimore, MD). cDNA was transcribed from 1 μg RNA using the Verso cDNA kit (Thermo Fisher Scientific, AB1453B) following the manufacturer’s instructions and the MiniAmp Thermal Cycler (Thermo Fisher Scientific) with the following parameters: 42°C for 30 min, 95°C for 2 min, and 4°C on hold.

### Quantitative PCR Analysis

Quantitative analysis of gene expression was performed using Taqman gene expression assays. Target mRNAs included TaqMan expression assays for genes related to neurotransmission (Table 1). Analysis was performed using the ABI QuantStudio 7 Flex Real-Time PCR System (Applied Biosystems, Carlsbad, CA) with the following parameters: 95°C for 10 min; 40 cycles of 95°C for 10 s and 60°C for 1 min. Gene expression was determined using the change in cycle threshold (ΔC_t_) method; with the difference between C_t_ values for the gene of interest and housekeeping gene for a given sample calculated as ΔC_t_ = (C_t_ gene of interest – C_t_ Rsp18). Data are expressed as means ± SD.

**Table 1.** Expression of genes associated with neurotransmission in adipose tissue

	aPVAT		mPVAT		WAT		Cortex	Region		Sex	
Gene ID	ΔCt female	ΔCt male	ΔCt female	ΔCt male	ΔCt female	ΔCt male	ΔCt female	*F*	*P* value	*F*	*P* value
*Ion channels, receptors, transporters*											
*Atplbl*	3.574 ± 0.227	4.579 ± 0.126	3.555 ± 1.140	4.283 ± 0.768	5.042 ± 0.352	4.945 ± 0.221	−2.2715	0.924	0.0528	2.513	0.1389
*Cacnlc*	8.38 ± 0.355^c,d^	8.987 ± 0.258^a,b^	7.386 ± 0.615	7.538 ± 0.190	7.356 ± 0.071	7.221 ± 0.185	3.1955	21.44	0.0001	1.203	0.2943
*Cacna2dl*	7.024 ± 0.676	7.963 ± 0.289	6.411 ± 0.563	6.475 ± 0.281	6.764 ± 0.135	6.635 ± 0.106	1.271	7.455	0.0079	1.58	0.2327
*Gria2*	12.999 ± 0.661	15.518 ± 1.165^e^	13.945 ± 0.317	12.910 ± 0.706^f,g^	16.645 ± 0.63 l^h,i^	15.505 ± 0.284	−0.204	15.32	0.0005	0.0818	0.7798
*Grik3*	12.181 ± 0.716	12.685 ± 0.531	11.617 ± 0.708	10.986 ± 0.385^j,k^	13.002 ± 0.279	13.277 ± 0.510	3.961	11.58	0.0016	0.0245	0.8781
*Grik5*	7.126 ± 0.850	7.572 ± 0.331	6.859 ± 0.597	6.983 ± 0.201	7.294 ± 0.128	7.466 ± 0.213	2.09	1.22	0.3292	0.8525	0.374
*Hcn4*	11.649 ± 0.171	12.496 ± 0.221^l,m^	11.332 ± 1.000	11.032 ± 0.402	9.637 ± 0.466^n,o^	10.128 ± 0.516	8.299	16.9	0.0003	1.25	0.2854
*Kcnab2*	13.038 ± 0.783^p,q^	13.594 ± 0.439^r,s^	9.040 ± 0.637	9.848 ± 0.085^t^	10.369 ± 0.436	11.733 ± 0.380^u^	3.059	58.46	<0.0001	9.581	0.0093
*Kcnip3*	8.401 ± 0.719	7.401 ± 0.246^v^	7.713 ± 0.662	8.110 ± 0.354	8.118 ± 0.198	7.800 ± 0.150	1.167	0.019	0.9811	1.41	0.2581
*Slc6a17*	11.631 ± 0.689	11.730 ± 0.297^w^	9.995 ± 0.740	9.470 ± 0.244	10.365 ± 0.336	10.440 ± 1.632	1.38	5.96	0.0159	0.0625	0.8068
*Adrala*	3.268 ± l.383^x,y^	3.585 ± 0.294^z,A^	7.070 ± 1.040	7.332 ± 0.399	5.699 ± 0.344	6.835 ± 0.442	4.345	26.1	<0.0001	1.654	0.2227
*Adrbl*	4.144 ± 1.639^B^	4.421 ± 0.497	6.364 ± 0.613	4.540 ± 0.574	6.686 ± 0.791	5.248 ± 0.875	2.9	3.558	0.0612	3.544	0.0842
*Adrb2*	4.389 ± 0.178	3.768 ± 0.391	4.767 ± 0.096	4.273 ± 0.767	4.934 ± 0.197	4.479 ± 0.217	5.748	2.645	0.1118	5.685	0.345
*Adrb3*	5.333 ± 1.027	5.526 ± 0.627^C^	4.609 ± 0.636	4.314 ± 0.185	3.960 ± 0.281	3.189 ± 0.251	12.327	10.19	0.0026	0.751	0.4031
*Chrml*	11.346 ± 0.674	12.201 ± 1.204	10.309 ± 0.242	10.747 ± 0.530	11.570 ± 0.155	10.693 ± 0.274	0.915	3.977	0.0473	0.148	0.7072
*Chrna7*	11.656 ± 0.295	12.244 ± 0.838	12.472 ± 0.529	11.878 ± 0.303	12.166 ± 0.395	13.143 ± 0.389	3.448	3.698	0.0561	1.287	0.2787
*Npylr*	11.497 ± 0.214^D,E^	11.352 ± 0.404^F^	9.938 ± 0.902	9.276 ± 0.134	10.018 ± 0.219	10.354 ± 0.304	6.358	17.51	0.0003	0.3753	0.5516
*Drdl*	11.862 ± 0.889^G,H^	12.494 ± 0.606^I,J^	9.426 ± 0.457	8.636 ± 0.324	9.726 ± 0.587	7.021 ± 0.381^K,L^	2.72	50.57	<0.0001	8.35	0.0136
*P2ry12*	14.073 ± 0.303^M,N^	13.615 ± 0.244	13.239 ± 0.343	13.134 ± 0.229	13.270 ± 0.176	13.127 ± 0.348	9.319	7.162	0.009	2.107	0.1723
*Synaptic vesicles*											
*Nlgnl*	9.298 ± 0.157^O,P^	9.112 ± 0.140^Q,R^	8.137 ± 0.397	7.526 ± 0.110^S^	7.799 ± 0.165	6.471 ± 0.264^T,U^	1.683	85.63	<0.0001	29.02	0.0002
*Syn*	6.336 ± 0.955	6.451 ± 0.358	7.138 ± 0.622	6.702 ± 0.143	6.538 ± 0.130	6.969 ± 0.386	−1.061	1.078	0.3711	0.0147	0.9054
*Synapse structure*											
*Gphn*	4.779 ± 0.526	5.487 ± 0.397	4.990 ± 0.527	4.646 ± 0.155	5.141 ± 0.132	4.534 ± 0.154	2.935	0.9632	0.4093	0.1511	0.7043
*SHANK1*	10.668 ± 0.046^V^	10.046 ± 0.265	9.165 ± 0.622	9.295 ± 0.207	9.376 ± 0.526	10.155 ± 0.886	0.031	4.878	0.0282	0.1058	0.7506
*Dlg4*	7.856 ± 0.558	7.724 ± 0.192	6.670 ± 0.523	6.424 ± 0.102	6.732 ± 0.116	6.604 ± 0.170	−0.376	16.72	0.0003	0.7573	0.4012

Values are means ± SD, with the degrees of freedom (F) and corresponding *P* value for two factors included in the statistical analyses: region and sex; *n* = 3 experimental males/females per region of adipose tissue, and *n* = 1 female for cortical tissue quantification of change in cycle threshold (ΔCt) values for all genes examined in male and female aortic mesenteric perivascular adipose tissue (aPVAT), aortic and mesenteric PVAT (mPVAT), white adipose tissue (WAT), and female cortical tissue. No statistical measures were performed using the cortical data generated from a signal female (*n* = 1). Statistical analysis comparing region and sex for each individual gene, not between genes, was performed using two-way ANOVA followed by Tukey’s multiple comparison test.

a *P* = 0.0023 vs. male mPVAT,

b *P* = 0.0005 vs. male WAT,

c *P* = 0.0224 vs. female mPVAT,

d *P* = 0.0264 vs. female WAT,

e *P* = 0.0034 vs. female aPVAT,

f *P* = 0.0070 vs. male aPVAT,

g *P* = 0.0072 vs. male WAT,

h *P* = 0.0005 vs. female aPVAT,

i *P* = 0.0055 vs. female WAT,

j *P* = 0.0225 vs. male aPVAT,

k *P* = 0.0032 vs. male WAT,

l *P* = 0.0447 vs. male mPVAT,

m *P* = 0.0022 vs. male WAT,

n *P* = 0.0072 vs. female aPVAT,

o *P* = 0.0206 vs. female mPVAT,

p *P* < 0.0001 vs. female mPVAT,

q *P* = 0.0006 vs. female WAT,

r *P* < 0.0001 vs. male mPVAT,

s *P* = 0.0085 vs. male WAT,

t *P* = 0.0079 vs. male mPVAT,

u *P* = 0.0200 vs. female WAT,

v *P* = 0.0453 vs. female aPVAT,

w *P* = 0.0405 vs. male mPVAT,

x *P* = 0.0009 vs. female mPVAT,

y *P* = 0.0208 vs. female WAT,

z *P* = 0.0010 vs. male mPVAT,

A *P* = 0.0031 vs. male WAT,

B *P* = 0.0412 vs. female WAT,

C *P* = 0.0045 vs. male WAT,

D *P* = 0.0111 vs. female mPVAT,

E *P* = 0.0153 vs. female WAT,

F *P* = 0.0014 vs. male mPVAT,

G *P* = 0.0029 vs. female mPVAT,

H *P* = 0.0074 vs. female WAT,

I *P* < 0.0001 vs. male mPVAT,

J *P* < 0.0001 vs. male WAT,

K *P* = 0.0379 vs. male mPVAT,

L *P* = 0.0005 vs. female WAT,

M *P* = 0.0294 vs. female mPVAT,

N *P* = 0.0359 vs. female WAT,

O *P* = 0.0007 vs. female mPVAT,

P *P* < 0.0001 vs. female WAT,

Q *P* < 0.0001 vs. male mPVAT,

R *P* < 0.0001 vs. male WAT,

S *P* = 0.0199 vs. female mPVAT,

T *P* = 0.0015 vs. male mPVAT,

U *P* < 0.0001 vs. female WAT, and

V *P* = 0.0306 vs. female mPVAT.

### Immunofluorescence Staining and Imaging

For all incubation and wash steps, tissue was protected from light and continually shaken on an orbital rocker at room temperature, exception incubation with the primary antibodies that were incubated at 4°C. Fixed adipose tissue was washed once for 10 min in 1× PBS, followed by a 10-min incubation with 0.4% Triton X-100/1× PBS (X100, Sigma) to permeabilize cells. The tissue was then washed twice for 10 min in 1× PBS and incubated for 1 h with blocking buffer [10% normal donkey serum (017-000-121, JacksonImmuno Research), 1% bovine serum albumin (BSA, 001-000-162, JacksonImmuno Research), 0.4% Triton X-100, 0.1% Tween-20 (P2287, Sigma); in 1× PBS]. Blocking buffer containing primary antibodies at a 1:100 dilution was added to the tissue and incubated for 3 days at 4°C (anti-rat CD31, Abcam, ab56299, RRID:AB_940884; Peripherin, Santa Cruz Biotechnology, sc-377093, RRID:AB_2923264). The tissue was then washed three times for 10 min in 1× PBS, followed by a 2-h incubation with secondary antibodies at room temperature (1:500 dilution; donkey anti-rat IgG Alexa 488, 712-545-150, RRID:AB_2340683; donkey anti-mouse IgG Alexa 594,715-585-150, RRID:AB_2340846, JacksonImmuno Research). Following three washes with 1× PBS, the tissue was incubated with 4′,6-diamidino-2-phenylindole dihydrochloride (DAPI, D8417, Sigma) for 30 min. After washing twice with 1× PBS for 10 min each wash, the tissue was mounted onto slides (16004-370, VWR) using Coverwell Imaging Chambers (70327-05, Electron Microscopy Sciences, Hatfield, PA) and hydromount (National Diagnostics, H5-106, Atlanta, GA). All mounted slides were stored in the dark at 4°C and imaged within a month of mounting.

Images were acquired through the HC PL APO CS2 ×10/0.40 dry objective using the Leica Stellaris 5 inverted confocal microscope (Leica, Deer Park, IL). Acquisition parameters relating to fluorescence intensity were optimized to each probe and tissue. Large area scans were obtained using the HC PL APO CS2 ×10/0.40 dry objective with the following parameters: 400 scan speed, line average of 2, 3- to 5-mm z-step, and 512 × 512 image resolution. Single frame images were obtained using either the HC PL APO CS2 ×10/0.40 dry objection, the HC PL APO CS2 ×20/0.74 dry objective, or the HC PL APO ×40/1.30 oil objective with a 400-scan speed, line average of 2–3, 1-mm z-step, and 1,024 × 1,024 image resolution. Images were analyzed using the Leica Stellaris LAS X software with final images being transferred to Adobe Illustrator (Adobe, San Jose, CA) for the construction of figure sets.

### Tissue Clearing

Fixed adipose tissue was cleared following the EZ Clear method described by Hsu et al. (51). Briefly, fixed adipose tissue was delipidated in 5 mL of 50% tetrahydrofurane (186562, Sigma) overnight at room temperature and washed four times for 1 h in ultrapure water. Delipidated adipose tissue then underwent immunofluorescence staining as outlined earlier in *Immunofluorescence Staining and Imaging* with an overnight blocking step in place of a 1-h block and four 1-h wash steps in place of 10-min wash steps. After the staining procedure was completed, the tissue was incubated overnight in EZ View sample mounting and imaging solution to clear (80% Nycodenz, Accurate Chemical & Scientific, 100334-594, Carle Place, NY), 7 M urea (Sigma, U5378), 0.05% sodium azide (Sigma, S2002), in 0.02 M sodium phosphate buffer. The cleared tissue was then mounted onto slides using EZ View sample mounting and imaging solution and Coverwell imaging chambers, taking care to remove any debris and bubbles. Mounted samples were then stored at room temperature in the dark and imaged within 3 wk.

### Calcium Imaging

Live tissue samples were visualized through a ×20 widefield water-immersion objective lens (1.0 numerical aperture, Olympus XLUMPFLN20xW, Center Valley, PA) on an upright Olympus BX51WI fixed-stage microscope (Center Valley, PA). Adipocytes were identified based on their spherical shape and expression of the fluorescent tdTomato (tdT) reporter protein. For aPVAT, white adipocytes overlaying the brown adipocytes were focused on for imaging as the brown adipocytes were too dense to image clearly. Imaging of calcium responses in the colon was performed to confirm effective EFS as the parameters used in this study induce maximal calcium responses in enteric neurons. The enteric nervous system was identified based on the expression of the tdTomato reporter protein as previously described (57). Fluorescent imaging illumination was supplied by a DG4 Xenon light source (Sutter Instrument, Novato, CA). The light used to excite GCaMP5g was filtered through a 485/20-nm band-pass filter, and emitted light was filtered through a 515-nm long-pass filter. The tdTomato was excited by light filtered through a 535/20-nm band-pass filter and filtered through a 610/75-nm band-pass emission filter before detection (4). Imaging data were acquired at a rate of 5 frames/s using a Neo sCMOS camera (Andor, South Windsor, CT) and MetaMorph software (v.7.10.1.161, Molecular Devices, San Jose, CA). Images were saved as .tiff files, and recordings were exported as .tiff stacks for further analysis. Throughout the experiment, bath application of 1× Kreb’s buffer via gravity perfusion at a rate of 2–4 mL/min (AutoMate Scientific, Berkeley, CA), heated to 37°C (TC-344C, Warner Instruments, Hamden, CT) was performed. Individual stimuli (10 mM Bethanechol, 10 mM NE) were applied for 30 s in a similar manner to the tissue with 5 min wash with 1× Kreb’s buffer (see *Drugs and Reagents* for complete formulation) in between. Electrical field stimulation (EFS) was performed following bethanechol and before NE stimulation as indicated in the following section.

### Electric Field Stimulation

Electric field stimulation (EFS) was used to stimulate broad neuronal depolarization within adipose and colonic tissue as previously described (57). *Adipoq^Cre+^;GCaMPtdT^f/WT^* aPVAT, mPVAT, and WAT was stimulated with a single pulse wave (+100 V, 0.1 ms, 10 Hz) generated from two platinum iridium wire electrodes (RC-27NE2, Warner Instruments, Hamden, CT) positioned on either side of the tissue prep connected to a voltage stimulator (Model 2100, A-M Systems, Carlsborg, WA). *Adipoq^Cre−^;RC::L-hM3Dq^f/WT^* mice were used to confirm and validate EFS in vascular imaging studies. These mice express a the Cre-recombinase-dependent hM3Dq allele with a FLEx switch cassette containing the eGFP sequence and an inverted hM3Dq/mCherry/2ACT88 fusion protein, under the control of the endogenous Gt(ROSA)26Sor promoter/enhancer regions and the CAG hybrid promoter. In the absence of the tissue-specific expression of Cre (driven by the Adiponectin promoter), the lox sites flanking the inverted hM3Dq/mCherry/2ACT88 fusion protein remain intact preventing the expression of the DREADD receptor and mCherry reporter protein, resulting in widespread expression of eGFP including the vasculature of adipose tissue. Single pulse waves (+100 V, 0.1 ms, 10 Hz; +100 V, 10 ms, 10 Hz) were administered to *Adipoq^Cre−^;RC::L-hM3Dq^f/WT^* mPVAT for confirmation of EFS by imaging and quantifying changes in vascular diameter, with EFS inducing vasoconstriction. *Wnt1^Cre+^;GCaMP5g-tdT^fl/WT^* mice were used to confirm and validate EFS in mesenteric nerves (*n* = 3 female mice) and colonic neurons (*n* = 1 male mouse). Single pulses waves (+100 V, 0.1 ms, 10 Hz; +100 V, 10 ms, 1 pulse) were administered to mPVAT and colonic circular muscle myenteric plexus preps and calcium responses were imaged and quantified as indicated in *Imaging and Data Analysis*. Gravity perfusion bath application of 1× Kreb’s buffer (*Drugs and Reagents*), warmed to 37°C, was supplied throughout the experiment.

### Vasculature Imaging

Mesenteric artery responses to EFS, serving as a positive control for EFS confirmation, were visualized in a similar manner to calcium imaging (*n* = 3 female mice). The artery in mPVAT was visualized using the same imaging setup and software as described earlier under *Calcium Imaging*. Vessel responses were imaged using an excitation light filtered through a 485/20-nm band-pass filter and emitted light filtered through a 515-nm long-pass filter. The artery was identified based on its linear morphology with a darker interior containing autofluorescing blood cells, confined by highly fluorescing parallel vessel walls on either side, and smaller size compared with the mesenteric vein. Gravity perfusion bath application of 1× Kreb’s buffer (see *Drugs and Reagents* for complete formulation), warmed to 37°C, was supplied throughout the experiment, except during application of NE. EFS and NE application were carried out as indicated earlier under *Calcium Imaging* and *Electrical Field Stimulation*.

### Data Analysis

Calcium recordings were analyzed using FIJI software (NIH, Bethesda, MD) as previously described (57). A tdTomato image obtained before beginning the experiment was used to confirm the identity of and location of adipocytes for all experiments, as well as to generate the regions of interest (ROIs) for subsequent analysis. Calcium responses in adipocytes to the various stimuli were quantified using the recordings obtained in the GFP channel. Final data values were quantified as the fold change in mean cellular fluorescence intensity relative to baseline florescence intensity (Δ*F*/*F*_0_) within a given adipocyte. Peak Δ*F*/*F*_0_ was used to visualize peak changes in adipocyte responses to bethanechol, EFS, and NE. A positive response was defined as a peak Δ*F*/*F*_0_ value > 3 SDs above the average baseline Ca^2+^ level measured within a cell for a fixed recording. Only cells within the field of view were used for analysis. Responses were measured from a total of 164–294 cells from three animals per stimuli. Percent responding cells was calculated as the number of cells with a positive response divided by the total number of cells multiplied by 100%. Data are expressed as means ± SD.

Successful EFS stimulation was confirmed in vasculature imaging and mesenteric nerve and colonic myenteric ganglion calcium responses. Change in mesentery artery, secondary branch (Fig. 1), diameter in response to EFS and NE was confirmed by measuring the distance (µm) between the inner artery walls using FIJI software. Representative images of mesenteric and colonic neuron responses to EFS (before and during) were obtained from calcium recordings and calcium responses (Δ*F*/*F*_0_) were quantified as indicated earlier.

### Statistical Analyses

Gene expression was analyzed by two-way ANOVA, followed by post hoc analysis using Tukey’s multiple comparison test. Calcium responses were analyzed by three-way ANOVA, followed by post hoc analysis using Tukey’s multiple comparison test. Percent cells responding to stimuli in ex vivo adipocyte calcium imaging was analyzed using the Fisher’s exact test. Data that did not pass normality were subjected to a cube root transformation for statistical analysis. Statistical analyses and graphic representations were performed using GraphPad Prism program v.10.0.2 for Windows (GraphPad Software, San Diego, CA) with a *P* < 0.05 being considered statistically significant.

## RESULTS

### Genes Encoding Synaptic Transmission Mechanisms Are Expressed in Perivascular and White Adipose Tissue

**Figure 2.**
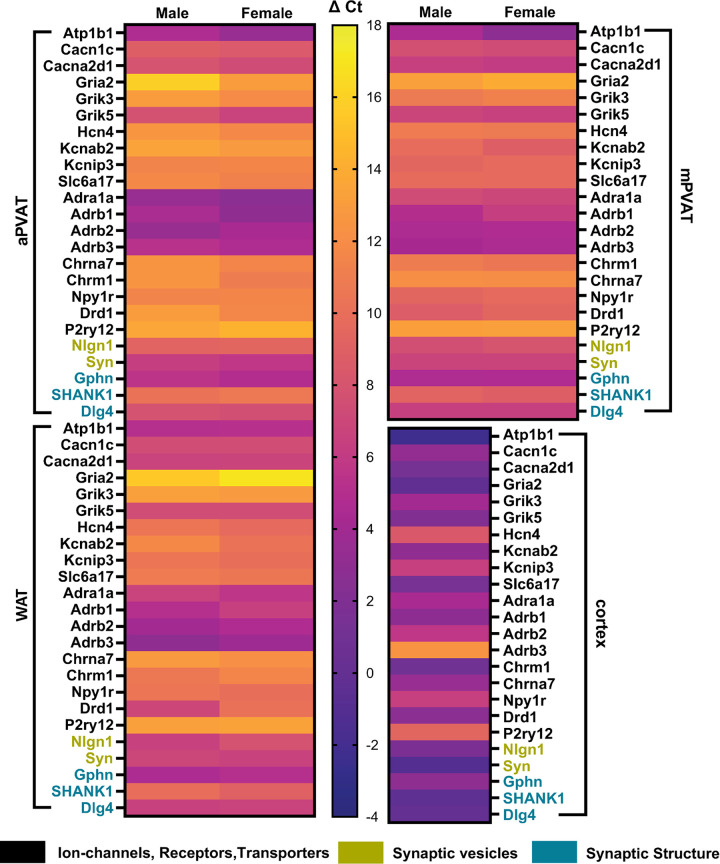
Expression of neurotransmission-associated gene in adipose tissue. Heat map illustrating relative expression levels for synaptic transmission-related genes in 3 regions of mouse adipose tissue: aortic perivascular adipose tissue (aPVAT), mesenteric perivascular adipose tissue (mPVAT), and white adipose tissue (WAT). Expression levels represented are the averaged change in cycle threshold (ΔCt) for each gene of interest (*n* = 3 per sex/adipose region). Selected genes included synaptic ion channels, receptors, and transporters (*Atp1b1, Cacn1c, Cacna2d1, Gria2, Grik3, Grik5, Hcn4, Kcnab2, Kcnip3, Slc6a17, Adra1a, Adrb1, Adrb2, Adrb3, Chrm1, Chrna7, Npy1r, Drd1, P2ry12*), as well as proteins involved in synaptic vesicle formation (*Nlgn1*, *Syn1*) and synapse structure (*Gphn*, *SHANK1*, *Dlg4*). Expression levels in a single female cortex were included as confirmation of qPCR. Color scale indicates relative gene expression levels, ranging from highest (ΔC_t_ = −4) to lowest (Δ*C_t_* = 18).

We began assessing the potential for neural innervation in PVAT by screening expression of genes associated with encoding mechanisms of synaptic signaling in three regions of adipose tissue: aPVAT, mPVAT, and WAT. Genes examined were selected from an RNAseq data set generated from Dahl S rat PVAT (58) and fell into three functional categories: *1*) ion channels, receptors, and transporters (*Atp1ba, Cacna1c, Cacna2d1, Gria2, Grik3, Grik5, Hcn, Kcnab2, Kcni3, Slcc6a17, Adra1a, Adrb1, Adrb2, Adrb3, Chrm1, Chrna7, Npy1r, Drd1, P2ry12*), *2*) synaptic vesicles (*Nlgn1, Syn*), and *3*) synapse structure (*Gphn, SHANK1, Dlg4*). Expression of these neurotransmission-related genes were detected in all three regions of adipose tissue (Fig. 2, Table 1). Of the 24 genes examined, only six showed a sex difference (Table 1). Cerebral cortex was included as a positive control (Fig. 2, Table 1) and as expected, exhibited high expression levels of synaptic genes. Therefore, gene expression data suggest the potential for neural innervation in PVAT and WAT, with differences in expression between regions and sexes.

### Nerve Fibers Present in PVAT and WAT Are Predominately Perivascular

**Figure 3.**
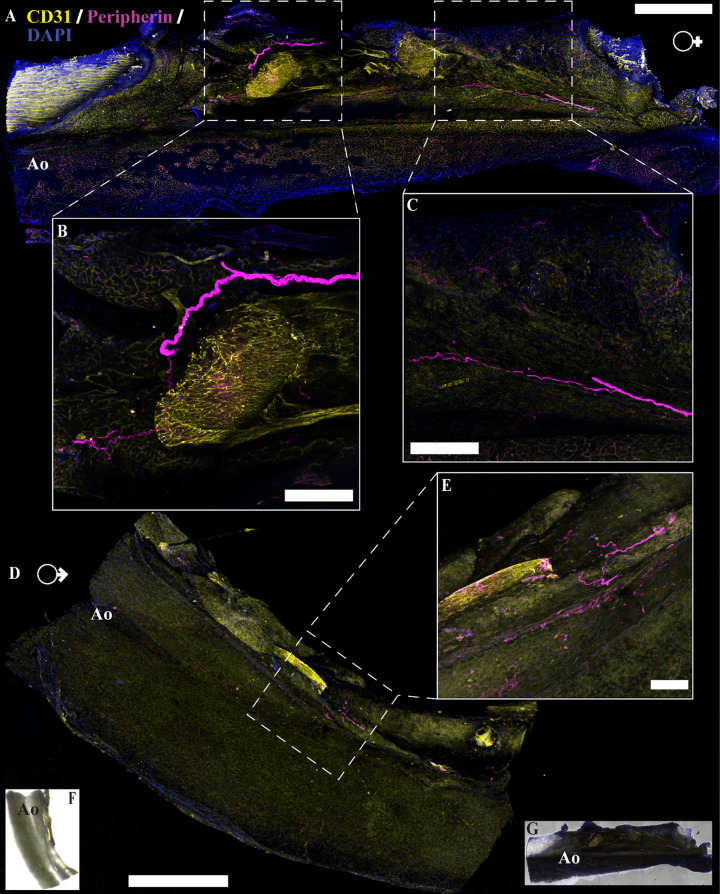
Neural innervation in female and male mouse aortic perivascular adipose tissue (aPVAT). Immunofluorescent labeling of neuron fibers (anti-peripherin, magenta), vasculature (anti-CD31, yellow), and nuclei [4′,6-diamidino-2-phenylindole dihydrochloride (DAPI), blue] in female and male aPVAT. *A*: representative confocal large area scan of stained female aPVAT (scale bar, 500 mm). *B* and *C*: ×10 images of labeled nerve fibers in female aPVAT (scale bar = 100 mm). *D*: representative large area scan of stained male aPVAT (scale bar = 500 mm). *E*: ×10 image of labeled nerve fibers in male aPVAT (scale bar = 100 mm). *F* and *G*: brightfield overlay of the large area scans for male (*F*) and female (*G*) mice. Ao, aorta. Representative of 3 females/males.

**Figure 4.**
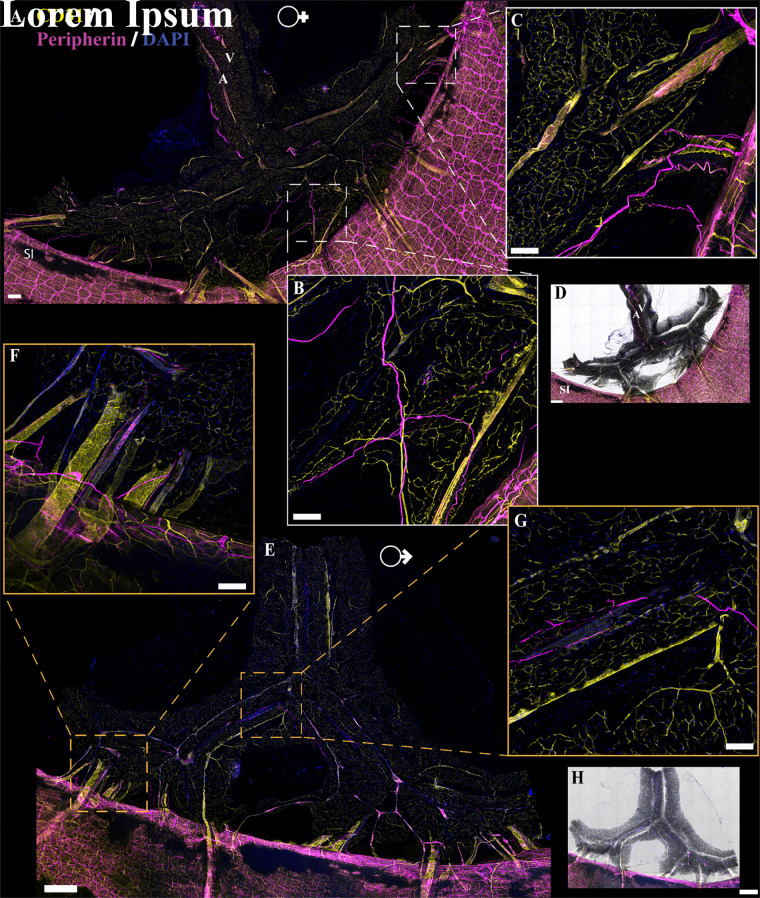
Innervation in female and male mouse mesenteric perivascular adipose tissue (mPVAT). Immunofluorescent labeling of neuron fibers (peripherin, magenta), vasculature (CD31, yellow), and nuclei [4′,6-diamidino-2-phenylindole dihydrochloride (DAPI), blue] in female and male mPVAT. *A*: representative confocal large area scan of stained female mPVAT (scale bar = 500 mm). *B* and *C*: ×10 images of labeled nerve fibers in female mPVAT (scale bar = 100 mm). *D*: brightfield overlay of stained female mPVAT (*A*). *E*: representative large area scan of stained male mPVAT (scale bar = 500 mm). *F* and *G*: ×10 images of labeled nerve fibers in male aPVAT (scale bar = 100 mm). *H*: brightfield overlay of the large area scan for male mPVAT. A, artery; SI, small intestines; V, vein. Representative of 3 females/males.

**Figure 5.**
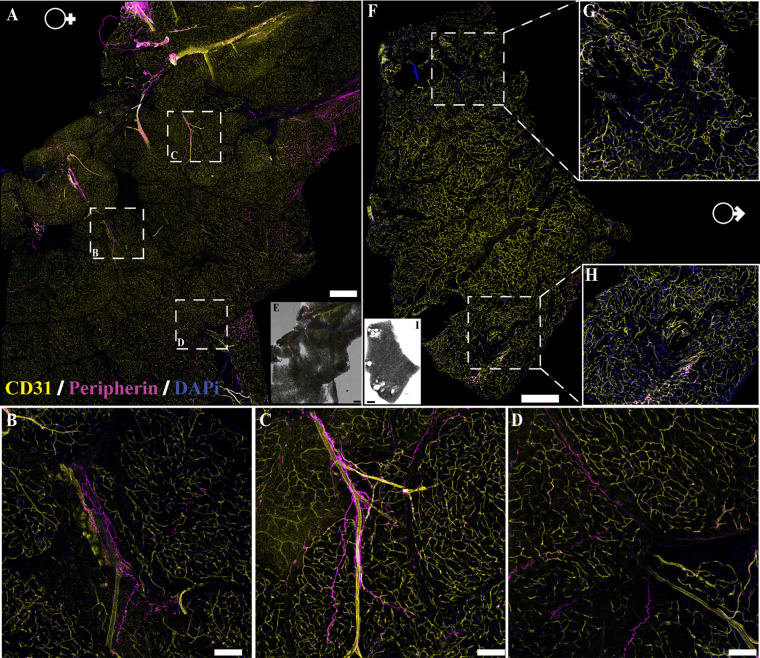
Innervation of white adipose tissue (WAT). Immunofluorescent labeling of neuron fibers (peripherin, magenta), vasculature (CD31, yellow), and nuclei [4′,6-diamidino-2-phenylindole dihydrochloride (DAPI), blue] in female and male WAT. *A*: representative confocal large area scan of stained female WAT (scale bar = 500 mm). *B–D*: ×10 images of labeled nerve fibers in female WAT (scale bar = 100 mm). *E*: brightfield overlay of the large area scan for female WAT (*A*). *F*: representative large area scan of stained male WAT (scale bar = 500 mm). *G* and *H*: ×10 images of labeled nerve fibers in male WAT (scale bar = 100 mm). *I*: brightfield overlay of the large area scan for male mesenteric perivascular adipose tissue (mPVAT). Representative of 3 females/males.

**Figure 6.**
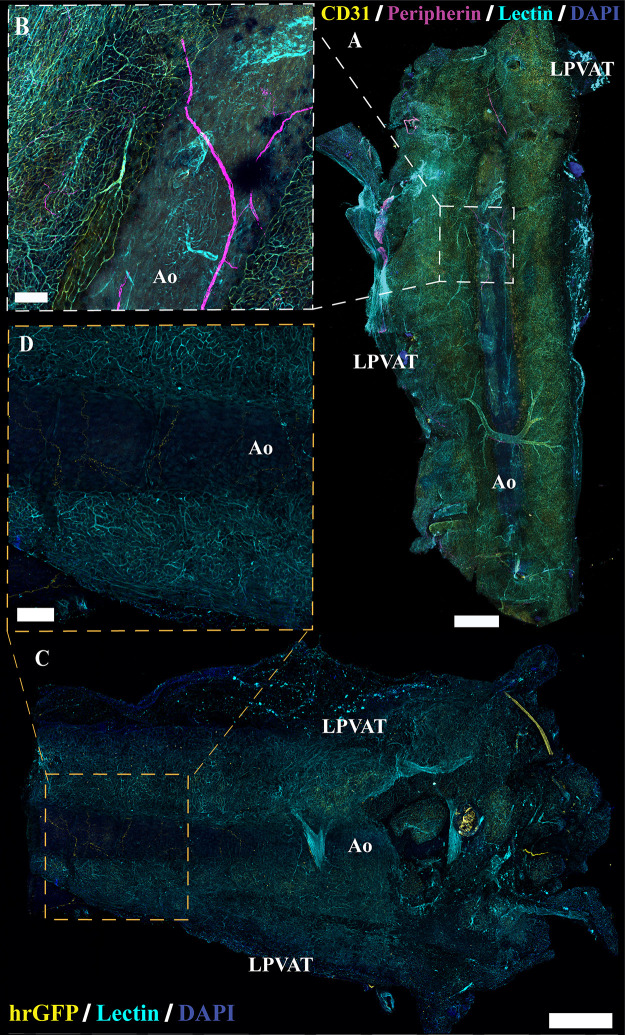
Visualization of innervation in cleared *Adipoq^Cre−^;GCaMP5g-tdT^fl/WT^* and noncleared *NPY-GFP* aortic perivascular adipose tissue (aPVAT). *A*: representative confocal large area scan of female *Adipoq^Cre−^;GCaMP5g-tdT^fl/WT^* aPVAT cleared and stained with anti-peripherin (magenta), anti-CD31 (yellow), lectin-649 (cyan), and 4′,6-diamidino-2-phenylindole dihydrochloride (DAPI, blue) (scale bar = 500 mm). *B*: ×10 image of a single frame within the large area scan (*A*) depicting labeled nerve fibers and vasculature (scale bar = 100 mm). Representative confocal large area scan of aPVAT from female *NPY-GFP* mice expressing transgenically labeled NPY nerve fibers (yellow) and stained with lectin (cyan) and DAPI (blue, scale bar = 500 mm). *D*: ×10 image of a single frame within the *NPY-GFP* large area scan (*C*) depicting labeled nerve fibers and vasculature (scale bar = 100 mm). Representative of 2 females for cleared female *Adipoq^Cre−^;GCaMP5g-tdT^fl/WT^* and 1 female for *NPY-GFP* mice. Ao, aorta; LPVAT, lateral PVAT.

A major tenet of the concept of innervation is the anatomical proximity of pre- and postsynaptic elements. Several recent reports argue that this requirement is fulfilled in WAT where sympathetic nerves are observed (43, 44, 46, 54). To determine if there is physical interaction between nerve fibers present within PVAT and the adipocytes themselves, we performed imaging studies using traditional immunofluorescence, tissue clearing, and transgenic mouse reporter lines. Traditional immunolabeling was used to identify nerves and vasculature within the tissue using antibodies against peripherin (pan-neuronal maker) and CD31 (vasculature marker). Similar patterns were observed in both male and female aPVAT (Fig. 3), mPVAT (Fig. 4), and WAT (Fig. 5). In large area confocal scans of stained aPVAT, peripherin-labeled nerve fibers were located along the aorta itself with minimal innervation in the PVAT in both females (Fig. 3, *A–C*) and males (Fig. 3, *D* and *E*). A similar distribution of nerve fibers was observed in large area scans of stained mPVAT with labeled nerves primarily residing alongside the secondary and tertiary branches of the mesenteric artery and vein, and little branching out into the adipocytes themselves in both females (Fig. 4, *A–C*) and males (Fig. 4, *E–G*). Comparable with aPVAT and mPVAT, nerves found within WAT were located appositional to the larger vasculature with fewer fibers reaching out into the adipocyte heavy regions of the tissue (Fig. 5). Although some WAT appeared to have more nerve fibers present in females (Fig. 5, *A–E*) than males (Fig. 6, *F–I*), this was not observed consistently. In addition, no notable alterations in amount or pattern of innervation were observed in male and female aPVAT or mPVAT (Figs. 3 and 4, respectively). Immunolabeling with additional antibodies (anti-P92, PGP9.5, anti-β-tubulin 3, l-noradrenaline) and dyes (NeuO) did not yield successful labeling of peripheral nerve fibers in adipose tissue (data not shown).

**Figure 7.**
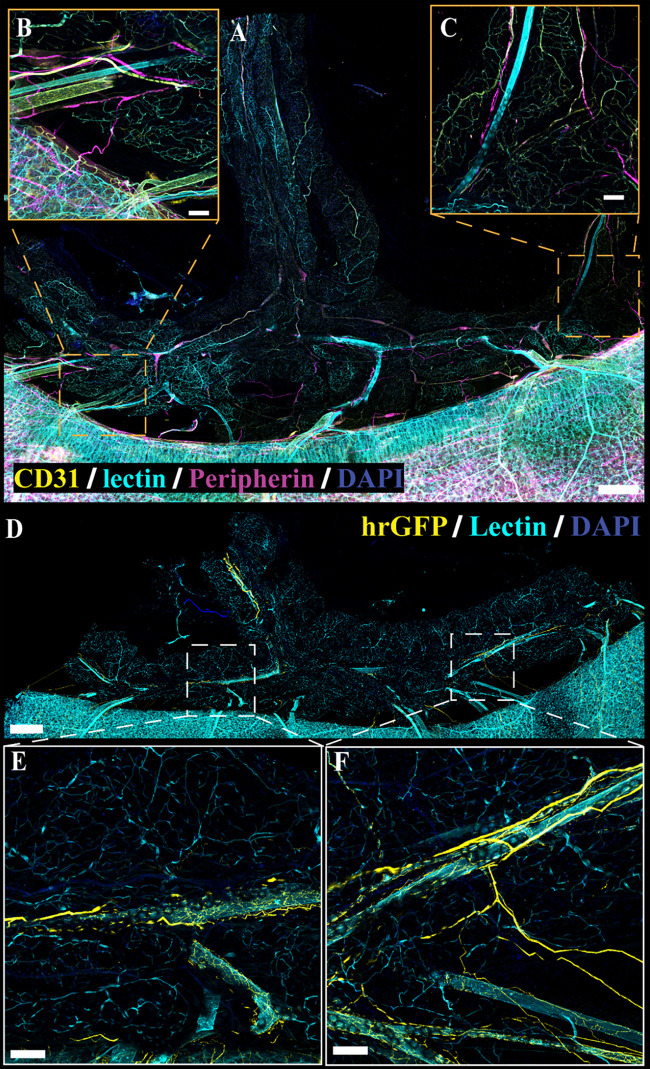
Innervation in cleared *Adipoq^Cre−^;GCaMP5g-tdT^fl/WT^* and noncleared *NPY-GFP* mesenteric perivascular adipose tissue (mPVAT). *A*: representative large area scan of cleared female *Adipoq^Cre−^;GCaMP5g-tdT^fl/WT^* mPVAT stained with anti-peripherin (magenta), anti-CD31 (yellow), lectin-649 (cyan), and 4′,6-diamidino-2-phenylindole dihydrochloride (DAPI, blue) (scale bar = 500 mm). *B* and *C*: ×10 images of a single frame within the cleared mPVAT large area scan (*A*) depicting labeled nerve fibers and vasculature (scale bar = 100 mm). *D*: representative large area scan of noncleared mPVAT from female *NPY-GFP* mice expressing transgenic labeling of NPY nerve fibers (yellow) and stained with lectin (cyan) and DAPI (blue, scale bar = 500 mm). *E* and *F*: ×10 images of a single frame within the *NPY-GFP* large area scan (*D*) depicting labeled nerve fibers and vasculature (scale bar = 100 mm). Representative of 2 females for cleared female *Adipoq^Cre−^;GCaMP5g-tdT^fl/WT^* and 1 female for *NPY-GFP*.

**Figure 8.**
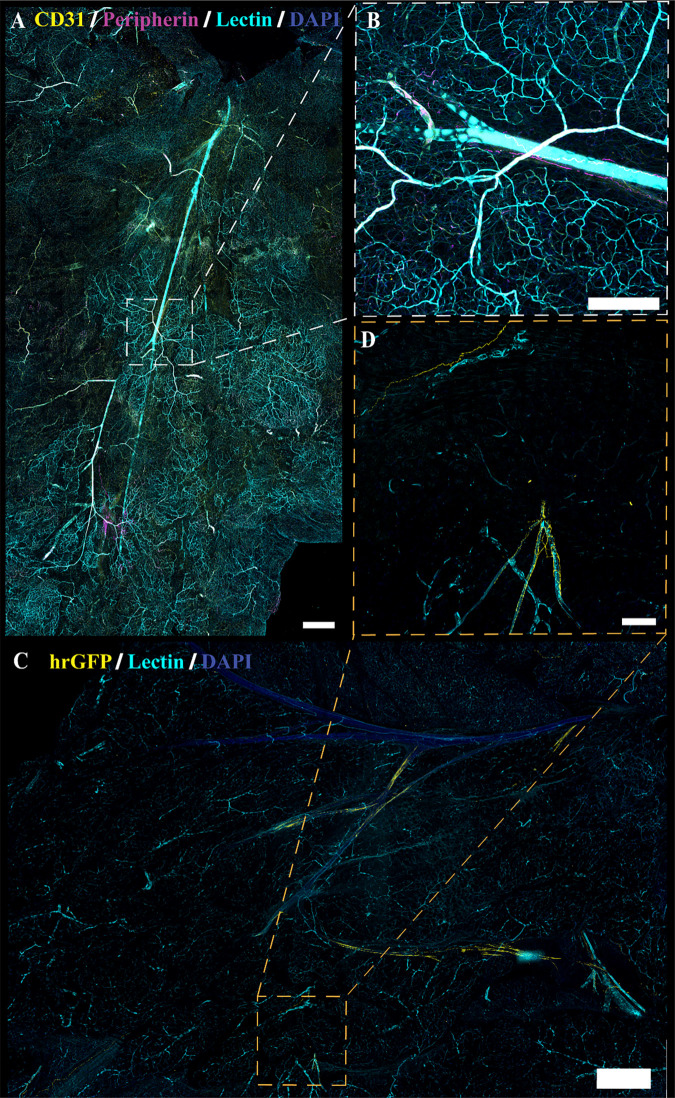
Neural innervation in cleared *Adipoq^Cre−^;GCaMP5g-tdT^fl/WT^* and noncleared *NPY-GFP* white adipose tissue (WAT). *A*: representative large area scan of cleared female *Adipoq^Cre−^;GCaMP5g-tdT^fl/WT^* WAT stained with anti-peripherin (magenta), anti-CD31 (yellow), lectin (cyan), and 4′,6-diamidino-2-phenylindole dihydrochloride (DAPI, blue) (scale bar = 500 mm). *B*: ×10 image of a single frame within the cleared WAT large area scan (*A*) depicting labeled nerve fibers and vasculature (scale bar = 100 mm). Representative large area scan of noncleared WAT from female *NPY-GFP* mice expressing transgenic labeling of NPY nerve fibers (yellow) and stained with lectin (cyan) and DAPI (blue, scale bar = 500 mm). *D*: ×10 image of a single frame within the *NPY-GFP* large area scan (*C*) depicting labeled nerve fibers and vasculature (scale bar = 100 mm). Representative of 2 females for cleared female *Adipoq^Cre−^;GCaMP5g-tdT^fl/WT^* and 1 female for *NPY-GFP*.

Immunolabeling and optical imaging in adipose tissue can be complicated by issues including poor antibody penetration and diffraction by lipids in adipocytes. In addition, while peripherin expression is robust in the autonomic neurons of the enteric nervous system, expression in other classes of peripheral nerve fibers is more variable (59, 60). For instance, within the dorsal root ganglion, not all neurons express peripherin (61). These findings suggest that labeling with peripherin, as with any single antibody, is an imperfect reflection of total nerve fiber content. To rule out the possibility that the lack of observed labeling was due to variations in the expression of target protein or technical issues in labeling, we conducted tissue clearing and staining combined with intravenous lectin injections to trace vasculature. Multiple clearing techniques were tested with the EZ Clear method producing the most robust results (51). Tissue clearing resulted in comparable labeling of nerves and vasculature in the aPVAT, mPVAT, and WAT (Figs. 6, *A* and *B*, 7, *A–C*, and 8, *A* and *B*, respectively) as observed in traditional immunolabeling (Figs. 3, 4, and 5). As there were no observable differences between males and females, only representative images from females are shown.

**Figure 9.**
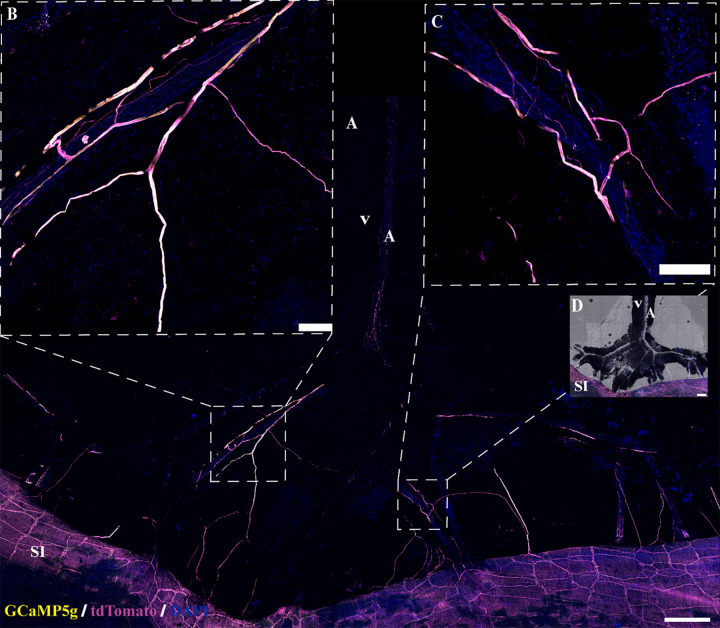
Neural innervation in mesenteric perivascular adipose tissue (mPVAT) in additional transgenic mouse models. Transgenic expression of fluorescent proteins in nerves present in the mPVAT in *Wnt1Cre^+^:GCaMPtdT^f/Wt^* female mice. Traditional immunofluorescence with 4′,6-diamidino-2-phenylindole dihydrochloride (DAPI) counterstaining and without vascular staining was used. *A*: representative confocal large area scan of mPVAT from *Wnt1Cre^+^:GCaMPtdT^f/Wt^* female mice expressing tdTomato and GCaMP5g expression in nerve fibers, counterstained with DAPI (scale bar = 500 mm, *n* = 3 females). *B* and *C*: ×10 images of single frames within the *Wnt1Cre^+^:GCaMPtdT^f/Wt^* large area scan depicting labeled nerve fibers and nuclei (scale bar = 100 mm). *D*: brightfield overlay of mPVAT obtained from transgenic *Wnt1Cre^+^:GCaMPtdT^f/Wt^* female mice expressing tdTomato and GCaMP5g in nerve fibers and counterstained with DAPI (*A*). A, artery; SI, small intestines; V, vein. Representative of 3 females.

Despite using antibodies considered as broad markers of peripheral nerves, we speculated that the limited extent of nerve fibers observed in immunolabeling experiments may be due to inefficient immunolabeling or lack of uniform expression target proteins within peripheral nerves (61). To circumvent this issue, we obtained several transgenic reporter lines targeting peripheral nerves and reassessed their anatomical location in PVAT and WAT. Regardless of promoter and reporter system, each line exhibited similar patterns of nerve fibers in adipose tissue as observed in immunolabeling experiments. As an example, aPVAT, mPVAT, and WAT were isolated from *NPY-GFP* mice, which were selected based on the efficacy of this line in marking sympathetic nerves and the role of NPY as a peptide neurotransmitter that is stored and released with NE from sympathetic nerve terminals (62–64). NPY^+^ nerves expressing humanized *Renilla* green fluorescent protein (hrGFP) were observed on the aorta and did not extend into the lateral PVAT of the aPVAT (Fig. 6, *C* and *D*). Similarly, in the mPVAT and WAT, NPY^+^ nerves resided largely along the vasculature with minimal spreading into the PVAT (Figs. 7, *D–F*, and 8, *C* and *D*, respectively). Genetic labeling did provide more extensive visualization of fine nerve fibers in the vascular nerve plexus in *NPY-GFP* mice that was not detectable with peripherin staining (Figs. 7, *E* and *F*, and 8*D*). Despite this, nerve fibers diverging from the vasculature and extending to contact adipocytes were rare. As there were no observable differences between males and females, only representative images from females are presented here. Adipose tissue from *Wnt1^Cre+^;GCaMP5g-tdT^f/WT^* and *Baf53^Cre+^;GCaMP5g-tdT^f/WT^* transgenic reporter mice also displayed similar anatomical patterns with nerve fibers contiguous to the large vasculature of the adipose tissue (Fig. 9, *A–D*, and data not shown, respectively). Other transgenic mouse reporter lines included in the study but not yielding successful imaging of adipose innervation were *TH*^Cre+^:*GCaMP5g-tdT^fl/WT^*, *TRPV1^Cre+^;GCaMP5g-tdT^fl/WT^*, and *Thy1-GFP* (data not shown).

**Figure 10.**
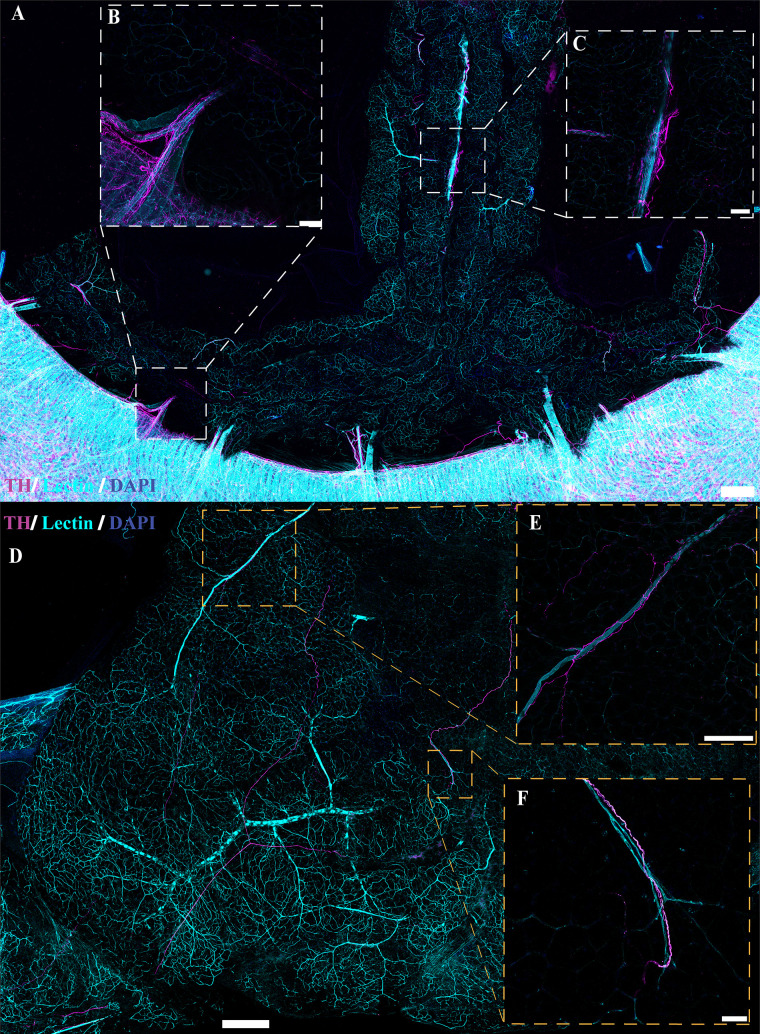
Sympathetic innervation in cleared *Adipoq^Cre−^;GCaMP5g-tdT^fl/WT^* mesenteric perivascular adipose tissue (mPVAT) and white adipose tissue (WAT). TH, tyrosine hydroxylase. *A*: representative confocal large area scan of cleared female *Adipoq^Cre−^;GCaMP5g-tdT^fl/WT^* mPVAT stained with anti-TH (magenta), lectin (cyan), and 4′,6-diamidino-2-phenylindole dihydrochloride (DAPI, blue) (scale bar = 500 mm). *B* and *C*: ×10 image of a single frame within the cleared mPVAT large area scan (*A*) depicting labeled nerve fibers and vasculature (scale bar = 100 mm). *D*: representative confocal large area scan of cleared female *Adipoq^Cre−^;GCaMP5g-tdT^fl/WT^* WAT stained with anti-TH (magenta), lectin (cyan), and DAPI (blue) (scale bar = 500 mm). *E*: ×10 image of a single frame within the cleared WAT large area scan (*D*) depicting labeled nerve fibers and vasculature (scale bar = 100 mm). *F*: ×20 image of a single frame within the cleared WAT large area scan (*D*) depicting labeled nerve fibers and vasculature (scale bar = 50 mm). Representative of 2 females.

**Figure 11.**
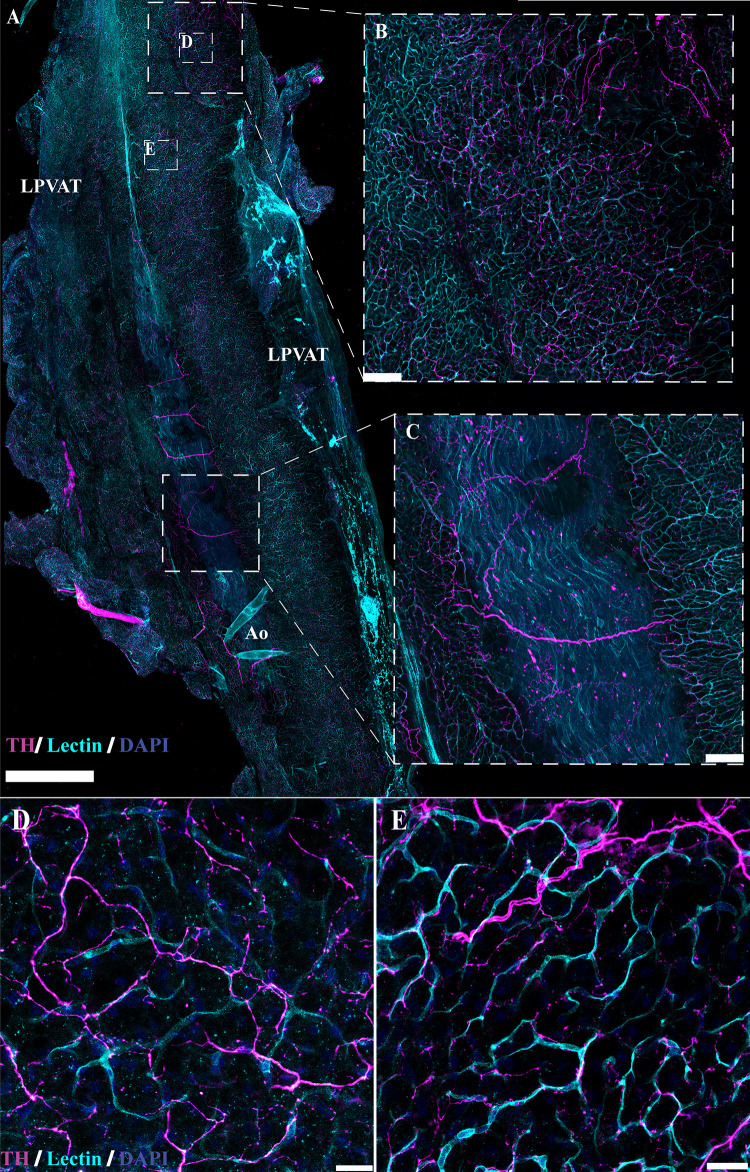
Sympathetic innervation in cleared *Adipoq^Cre−^;GCaMP5g-tdT^fl/WT^* aortic perivascular adipose tissue (aPVAT). TH, tyrosine hydroxylase. *A*: representative confocal large area scan of cleared female *Adipoq^Cre−^;GCaMP5g-tdT^fl/WT^* aPVAT stained with anti-TH (magenta), lectin (cyan), and 4′,6-diamidino-2-phenylindole dihydrochloride (DAPI, blue) (scale bar = 1,000 mm). *B* and *C*: ×10 image of a single frame within the cleared aPVAT large area scan (*A*) depicting labeled nerve fibers and vasculature (scale bar = 100 mm). *D* and *E*: ×40 image of a single frame within the cleared aPVAT large area scan (*A*) depicting labeled nerve fibers and vasculature (scale bar = 25 mm). Ao, aorta; LP, lateral PVAT. Representative of 2 females.

Several studies have indicated the presence of sympathetic innervation in adipose tissue (42, 43, 46, 47, 49, 50, 65–71). To confirm these findings, immunolabeling with anti-tyrosine hydroxylase (TH) in optically cleared tissue was also performed to examine sympathetic innervation in adipose tissue (Figs. 10 and 11). Similar to the results observed with peripherin immunolabeling and genetic labeling of NPY and Wnt1 neurons, innervation of TH-immunoreactive nerve fibers was limited and located predominantly adjacent to the larger vasculature in mPVAT and WAT with minimal branching into the adipose itself (Fig. 10, *A–C* and *D–F*, respectively). In contrast, TH-immunoreactive nerve fibers were extensive throughout the aPVAT (Fig. 11) as compared with the mPVAT and WAT (Fig. 10). TH-labeled nerves were observed surrounding the aorta and extending into the lateral PVAT of the aPVAT (Fig. 11, *A–C*). Higher magnification imaging revealed that these fibers were still located predominantly adjacent to the vasculature rather than extending and intertwining with individual adipocytes within the aPVAT (Fig. 11, *D* and *E*). Thus, while the aPVAT exhibited a higher degree of sympathetic innervation than the mPVAT and WAT as indicated by TH-labeling of nerve fibers, the location of these fibers in all adipose depots remains vessel-centric, in agreement with previous labeling and imaging.

### Norepinephrine Induces Calcium Responses in Adipocytes

**Figure 12.**
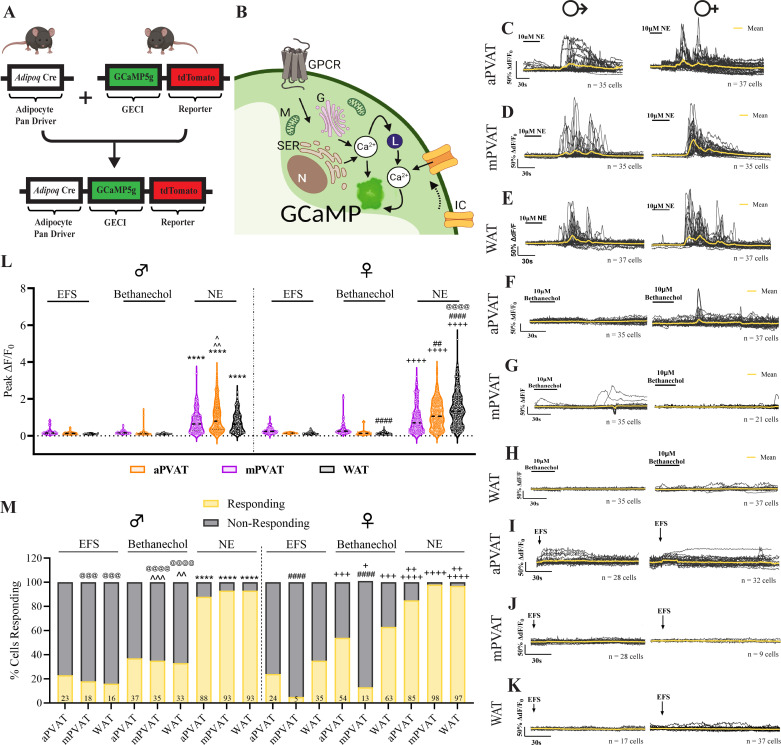
Adipocytes are not functionally regulated by neural input. *A*: *Adipoq^Cre+^;GCaMPtdT^f/Wt^* mice express the genetically encoded calcium indicator, GCaMP5g, and reporter protein, tdTomato, under control of the adipocyte pan driver, adiponectin. *B*: schematic depicting adipocyte activation involving increased cytoplasmic Ca^2+^ release from G protein-coupled receptor or ion channel activation. *C–E*: representative traces of male and female adipocyte responses (Δ*F*/*F*_0_) to bath application of 10 mM norepinephrine (NE) in aortic perivascular adipose tissue (aPVAT), mesenteric perivascular adipose tissue (mPVAT), and white adipose tissue (WAT), respectively (*n* = 17–37 cells). *F–H*: representative traces of male and female adipocyte responses to bath application of 10 mM bethanechol in aPVAT, mPVAT, and WAT, respectively (*n* = 21–37 cells). *I–K*: representative traces depicting male and female adipocyte responses to electrical field stimulation (EFS) (+100 V, 0.1 ms, 10 Hz, 1 pulse) in aPVAT, mPVAT, and WAT, respectively (*n* = 9–37 cells). *L*: quantification of peak adipocyte responses (peak Δ*F*/*F*_0_) to electrical field stimulation (EFS), bethanechol, and NE in male and female aPVAT, mPVAT, and WAT (*n* = 164–294 cells from 3 animals per experimental group). *M*: percentage of cells responding to EFS, bethanechol, and NE stimulation in male and female aPVAT, mPVAT, and WAT (*n* = 164–294 cells from 3 animals per experimental group). Statistical analyses was as follows: *L*: 3-way ANOVA, Tukey’s post hoc: ****P < 0.0001, male aPVAT NE vs. male aPVAT EFS and male aPVAT bethanechol, male mPVAT NE vs. male mPVAT EFS and male mPVAT bethanechol, male WAT NE vs. male WAT EFS and male WAT bethanechol. ^*P* = 0.0241, male mPVAT NE vs. male aPVAT NE. ^^*P* = 0.0021, male mPVAT NE vs. male WAT NE; ++++*P* < 0.0001 female aPVAT NE vs. female aPVAT EFS and female aPVAT bethanechol, female mPVAT NE vs. female mPVAT EFS and female mPVAT bethanechol, female WAT NE vs. female WAT EFS and female WAT bethanechol. ##*P* = 0.0011, female mPVAT NE vs. female aPVAT NE. ####*P* < 0.0001, female WAT NE vs. female aPVAT NE and female mPVAT NE, female aPVAT bethanechol vs. female WAT bethanechol. @@@@*P* < 0.0001, female WAT NE vs. male WAT NE. *M*: Fisher’s exact test, ^^*P* = 0.0015, male WAT EFS vs. male WAT bethanechol. ^^^*P* = 0.0002, male mPVAT EFS vs. male mPVAT bethanechol. ****P < 0.0001, male aPVAT NE vs. male aPVAT EFS and male aPVAT bethanechol, mPVAT NE vs. male mPVAT EFS and male mPVAT bethanechol, male WAT NE vs. male WAT EFS and male WAT bethanechol. +*P* = 0.0377, female mPVAT EFS vs. female mPVAT bethanechol. ++*P* = 0.0094, female aPVAT bethanechol vs. female aPVAT NE, ++*P* = 0.0015 female WAT bethanechol vs. female WAT NE, +++*P* = 0.0002 female aPVAT EFS vs. female aPVAT bethanechol, female WAT EFS vs. female WAT bethanechol; ++++*P* < 0.0001 female aPVAT NE vs. female aPVAT EFS bethanechol, female mPVAT NE vs. female mPVAT EFS and female mPVAT bethanechol, female WAT NE vs. female WAT EFS; ####*P* < 0.0001 female mPVAT EFS vs. female aPVAT EFS and female WAT EFS, female mPVAT bethanechol vs. female aPVAT bethanechol and female WAT bethanechol; @@@@*P* < 0.0001 male mPVAT bethanechol vs. female mPVAT bethanechol, male WAT bethanechol vs. female WAT bethanechol, @@@*P* = 0.0006 male WAT EFS vs. female WAT EFS, @@@*P* = 0.0001 male WAT bethanechol vs. female WAT bethanechol. G, Golgi; GECI, genetically encoded Ca^2+^ indicator; GPCR, G protein-coupled receptor; IC, ion channel; L, Lysosome; Lu, lumen; M, mitochondria; N, nucleus; Ser, smooth endoplasmic reticulum; VW, vessel wall. Images created with a licensed version of BioRender.com.

**Figure 13.**
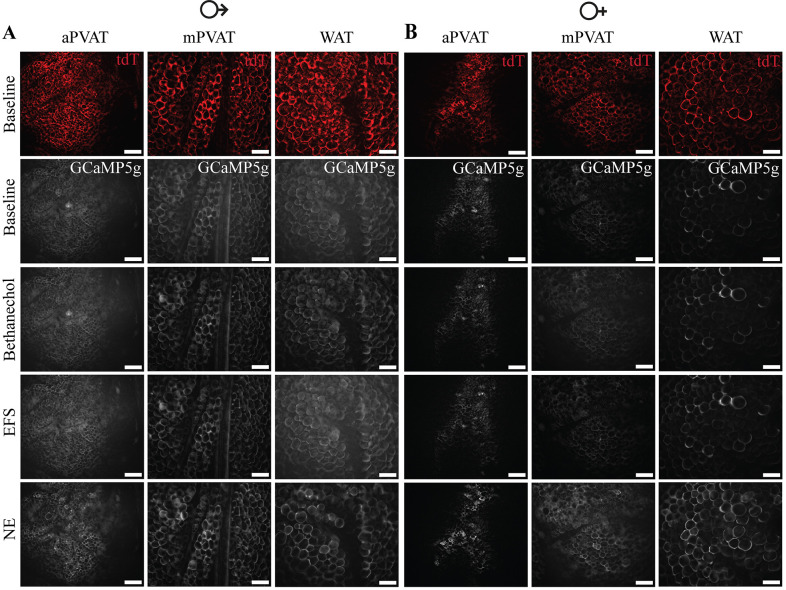
Transgenic expression of reporter protein, tdTomato (tdT), and genetically encoded calcium indicator, GCaMP5g, in adipocytes of *AdipoqCre^+^;GCaMPtdT^f/Wt^* transgenic mice. *A* and *B*: representative images of aortic perivascular adipose tissue (aPVAT), mesenteric perivascular adipose tissue (mPVAT), and white adipose tissue (WAT) from male (*A*) and female (*B*) *AdipoqCre^+^;GCaMPtdT^f/Wt^* mice demonstrating the expression of the reporter protein tdTomato and the genetically encoded calcium indicator, GCaMP5g, in mesenteric adipocytes (scale bar = 100 µm). Representative images of calcium responses in male (*A*) and female (*B*) mice to stimulation with 10 µM bethanechol, electrical field stimulation (EFS, +100 V, 0.1 ms, 10 Hz, 1 pulse), and 10 µM norephinephrine (NE).

To be considered “innervated,” the postsynaptic cell must exhibit responsiveness to transmitters released by the presynaptic cell. As our molecular analysis indicated the presence of genes associated with neurotransmission in adipose tissue, while imaging studies found limited innervation around adipocytes themselves, we performed live-cell calcium imaging to determine if adipocytes could be functionally regulated by nerve fibers present in adipose tissue. Norepinephrine has been implicated in driving adipocyte release of anticontractile factors, so we began by testing adipocyte responsiveness to this classical sympathetic transmitter (35, 42). To this end, we performed ex vivo live-cell calcium imaging on aPVAT, mPVAT, and WAT isolated from *Adipoq^Cre+^;GCaMP5g-tdT^f/WT^* mice. Adipocytes from this transgenic reporter mouse line express the genetically encoded calcium indicator, GCaMP5g, and reporter protein, tdTomato (Figs. 12, *A* and *B*, and 13, *A* and *B*). Calcium responses in adipocytes, indicated by the change in intensity of GCaMP5g fluorescence, were quantified before, during, and following bath application of 10 µM norepinephrine. Norepinephrine stimulation produced robust adipocyte responses in all experimental groups with regional differences within sex (Figs. 12, *C–E*, and *L*, and 3, *A* and *B*). Male mPVAT had significantly higher peak responses (peak Δ*F*/*F*_0_) than male aPVAT and WAT (Fig. 12*L*). Female aPVAT showed significantly lower peak responses compared with female mPVAT and WAT, with female WAT also exhibiting significantly higher peak responses compared with female mPVAT (Fig. 12*L*). Differential responses to norepinephrine were found between the sexes in the WAT. Norepinephrine stimulation resulted in significantly higher peak calcium responses in female WAT compared with male WAT with no significant differences observed between male and female aPVAT and mPVAT (Fig. 12*L*). Corresponding to peak calcium responses, the number of cells responding to norepinephrine was robust in both males and females, but no regional or sex differences were found in the number of cells responding (Fig. 12*M*). These results show that adipocytes in PVAT and WAT are responsive to norepinephrine, suggesting that they could respond norepinephrine release from sympathetic nerves.

### Adipocytes Display Minimal Responsiveness to Bethanechol

Parasympathetic postganglionic nerves and some sympathetic postganglionic neurons are cholinergic. To determine if adipocytes could respond to cholinergic neuronal signaling, we stimulated samples of aPVAT, mPVAT, and WAT from *Adipoq^Cre+^;GCaMP5g-tdT^f/WT^* mice with the muscarinic cholinergic agonist, bethanechol (10 mM). Bethanechol is an FDA-approved muscarinic ester that mimics the actions of acetylcholine, a neurotransmitter released in the sympathetic nervous system in intermediate steps and as the final chemical messenger of parasympathetic nervous system, while not subject to degradation by acetylcholine esterase. Adipocyte calcium responses (peak Δ*F*/*F*_0_) evoked by bethanechol (Figs. 12, *F–H*, and 13, *A* and *B*) were minimal compared with responses induced by norepinephrine for all three regions of adipose tissue in both sexes (Fig. 12*L*). Only one regional difference in bethanechol response was found between female WAT and female aPVAT. No sex differences were detected in male and female adipose tissue in response to bethanechol (Fig. 12*L*). Corresponding to peak calcium responses, the percentage of adipocytes responding to bethanechol were appreciably lower than those responding to norepinephrine (Fig. 12*M*). However, unlike the peak calcium responses, regional and sex differences were observed in response to bethanechol in terms of the number of responding cells. One regional difference was observed in females with significantly fewer cells responding to bethanechol stimulation in the mPVAT compared with the aPVAT and WAT (Fig. 12*M*). In addition, differences in the percentage of cells responding to bethanechol varied significantly between male and female mPVAT and WAT with fewer responding cells in the female mPVAT compared with the male mPVAT and more responding cells in the female WAT compared with the male WAT (Fig. 12*M*). The small proportion of adipocytes categorized as bethanechol-responding despite the minimal response evoked is likely due to two factors: the responses being only slightly above the established threshold of three standard deviations above baseline and the potential expression of muscarinic receptors in adipocytes (Table 1) (72, 73). Thus, the data suggest a minimal role as a potential mediator of nerve-adipocyte interactions.

### Nerve Fiber Depolarization Evokes Minimal Activity in Adipocytes

A fundamental criterion of innervation is that responses in the presynaptic cell evoke responses in the postsynaptic cell. The ability of nerve fibers residing within adipose tissue to modulate adipocyte activity was assessed using ex vivo live-cell calcium imaging and EFS (+100 V, 0.1 ms, 10 Hz). aPVAT, mPVAT, and WAT used were identical adipose tissue pieces to those isolated and subjected to NE and bethanechol stimulation. Despite the strong EFS stimulus applied, EFS induced minimal adipocyte responses compared with norepinephrine (Figs. 12, *I–K*, and 13, *A* and *B*) in all regions and sexes examined. As with bethanechol, peak calcium responses in adipocytes following EFS were considerably lower than those observed following NE in both sexes, across all adipose regions (Fig. 12*L*). No regional or sex differences were observed in male and female adipose tissue in response to EFS (Fig. 12*L*). In accordance with peak response data, the percentage of cells responding to EFS was notably lower for all experimental groups compared with norepinephrine (Fig. 12*M*). A regional difference was found with the number of responding cells being significantly lower in the mPVAT in females compared with the aPVAT and WAT (Fig. 12*M*). The number of cells responding to EFS were found to differ significantly between male and female mPVAT and WAT, with fewer cells responding in female mPVAT to EFS and more cells responding to EFS in female WAT than their male counterparts (Fig. 12*M*). The small proportion of adipocytes categorized as responding to EFS despite the minimal response evoked is likely due to the responses being only slightly above the established threshold of three standard deviations above baseline and the potential expression of voltage-gated ion channels in adipocytes that could be activated independently by EFS (74–76). These observations show that activating nerve fibers present in adipose tissue does not elicit subsequent responses in adipocytes to an extent that would suggest complete innervation, but that rather only a limited number of adipocytes are innervated. Furthermore, these results, combined with norepinephrine and bethanechol stimulation data, highlight differences in how adipocytes respond to stimuli depending on their anatomical location and biological sex.

**Figure 14.**
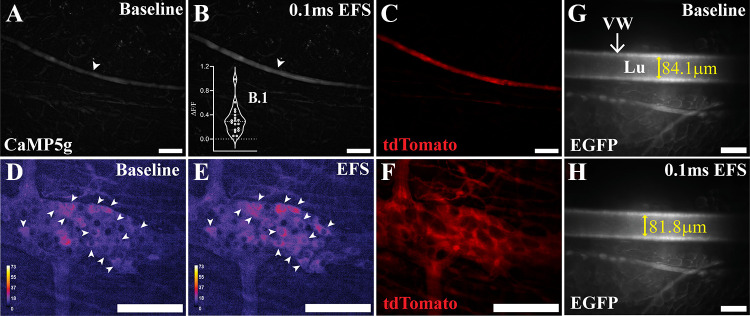
Confirmation of electrical field stimulation. *A–C*: confirmation of electrical field stimulation (EFS) by imaging nerve fiber responses in the mesenteric perivascular adipose tissue (mPVAT) from *Wnt1^Cre+^;GCaMPtdT^f/WT^* mice (*n* = 3 males). *A*: representative image of baseline calcium activity in nerve fibers (white arrowhead) in the mPVAT (scale bar = 100 µM). *B*: representative image of nerve fiber responses to EFS (+100 V, 0.1 ms, 10 Hz, scale bar = 100 µM). *C*: representative image of tdTomato expression in nerves within the mPVAT of *Wnt1^Cre+^;GCaMPtdT^f/WT^*. *D* and *F*: confirmation of EFS by imaging neuron responses in the colon from *Wnt1^Cre+^;GCaMPtdT^f/WT^* mice (*n* = 1 female). *D*: baseline calcium activity in neurons (white arrowheads) in the circular myenteric plexus (scale bar = 100 mm). *E*: response of neurons to EFS (+100 V, 0.1 ms, 10 Hz, scale bar = 100 mm). Color scale indicates intensity of responses. *F*: expression of reporter protein tdTomato in neurons (scale bar = 100 mm). *G* and *H*: confirmation of EFS in enhanced green fluorescent protein (EGFP)-labeled vasculature in mPVAT from *Adipoq^Cre+^;RC-L-hMD3q^f/Wt^* mice (*n* = 3 females). *G*: representative measurement of artery diameter in unstimulated mPVAT (84.1 mm, scale bar = 100 mm). *H*: representative decrease in artery diameter in response to EFS (+100 V, 0.1 ms, 10 Hz; 81.8 mm; scale bar = 100 mm).

The electrical stimulus applied to evoke activity in nerves traversing PVAT was strong; however, we sought additional confirmation to ensure that the stimulus evokes maximal responses in neurons. EFS stimulation induces neuronal calcium responses in the colonic myenteric plexus (57) and vasoconstriction in blood vessels (77). To independently confirm the efficacy of the electrical stimulus applied, we monitored neuronal calcium responses in the colonic myenteric plexus and the mPVAT and measured changes in mesenteric artery diameter. Electrical stimulation induced responses in nerve fibers in the mPVAT from *Wnt1^Cre+^;GCaMP5g-tdT^f/WT^* mice with identical parameters of EFS as administered to adipose tissue (white arrowheads, Fig. 14, *A–C*). EFS induced a 0.314 ± 0.219 Δ*F*/*F*_0_ response in mesenteric nerve fibers compared with baseline (Fig. 14, *B* and *B.1*). As expected, stimulation of colonic tissue from *Wnt1^Cre+^;GCaMP5g-tdT^f/WT^* mice with identical parameters of EFS as administered to adipose tissue, resulted in neuronal activation in myenteric ganglia (white arrowheads, Fig. 14, *D* and *E*). *Adipoq^Cre–^;RC::L-hM3Dq^f/WT^* transgenic mice express EGFP in all cells in the absence of Cre, including the vasculature of adipose tissue, which enabled clear visualization of adipose blood vessels. Electrical stimulation of mPVAT resulted in a 2.8 ± 0.7 mm decrease in the diameter of the secondary branch of the mesentery artery (Fig. 14, *G* and *H*). Further increasing the pulse duration to 10 ms from 0.1 ms resulted in a 32.8 ± 7.6 mm decrease in vessel diameter (data not shown). In addition, exposure of the same mPVAT to bath application of 10 mM norepinephrine to confirm vessel responses to stimuli, resulted in vasoconstriction of the mesentery artery, with a decrease of 48.1 ± 2.9 mm in diameter (data not shown). These data confirm that the EFS stimulus applied was sufficient to evoke broad neuronal depolarization among peripheral neurons and therefore, the lack of adipocyte response to EFS is due to a limited innervation rather than a technical issue.

## DISCUSSION

Neural innervation of adipose tissue is a concept that has evolved since the early 1960s and involves potential lipomotor neurons in the sympathetic chain (66, 78). This longstanding notion combined with the observation that norepinephrine, a sympathetic transmitter, stimulates the anticontractile effects of PVAT led to the assumption that PVAT is innervated by sympathetic neurons. Here, we formally test this concept using contemporary anatomical and functional approaches. Surprisingly, we find that nerve fibers traversing PVAT and WAT are predominantly associated with the vasculature and do not exhibit anatomical connections with adipocytes suggestive of innervation. In agreement, functional experiments show that maximal stimuli applied to nerves in PVAT elicit minimal responses in adipocytes, despite their high responsiveness to norepinephrine. Therefore, the combined data suggest that PVAT does not receive significant classical innervation as observed in other tissues.

Our findings regarding neural innervation in adipose tissue highlight an important distinction between physical and functional, or classical, innervation of tissue. Classical innervation of tissue requires three main components: *1*) the presence of nerves within the tissue, *2*) the presence of functional synapses between nerves and target cells within the tissue, and *3*) the ability of the nerves present within the tissue to control the function of its target cells (79). Therefore, for PVAT to meet the criteria to be considered classically innervated it would have to possess nerves that form functional synaptic connections with adipocytes or other tissue resident cells and control adipocyte function or the function of other resident target cells. PVAT and WAT are innervated with sympathetic and sensory nerve fibers; however, the extent of this innervation is variable (43, 44, 46, 48, 49, 54). In agreement, we also observed nerve fibers within PVAT and WAT. However, in contrast to studies demonstrating dense innervation of adipose tissue, our findings found only a dense innervation of sympathetic nerves in the aPVAT and not the mPVAT and WAT. In addition, our findings suggest nerve fibers, regardless of density, are located mainly appositional to vasculature within adipose tissue. Thus, regarding the first criteria, our results demonstrate that while nerve fibers are present, they are not fully distributed through the adipose itself, suggesting limited innervation.

The second criterion requires the presence of functional synaptic connections between nerve fibers and adipocytes. Evidence for the presence of neural presynaptic terminal and pre-/postsynaptic connections between nerves and target cells is limited. Although the expression of genes associated with encoding neurotransmission within adipose tissue has been confirmed, including in this study, the physical presence of synapses and precise distribution of these elements have not been fully explored (58, 80, 81). Imaging carried out to define innervation of PVAT and WAT, including in this study, relied on imaging techniques that preclude the nanoscale detection of synaptic terminals (43, 46, 48, 56, 82). Thus, innervation within the PVAT has been established on a larger micron scale, without addressing whether these innervating fibers establish synaptic connections with target cells or if they are simply passing through the adipose tissue (“en passant”). Although a mention of synaptic terminal presence in adipose tissue has been reported, subsequent studies have not expanded upon or recapitulated this result (83). Thus, the presence, localization, and number of synaptic terminals within PVAT is not yet fully authenticated. Further studies using superresolution imaging techniques such as electron microscopy, photoactivated localization microscopy, or stochastic optical reconstruction microscopy, should be conducted to better address the presence, distribution, and target cell population of synaptic terminals in PVAT.

Adipocyte responsiveness to neurotransmitters and the bioactive role of PVAT in modulating vascular tone suggest that PVAT is functionally innervated (27, 28, 37, 38, 42, 44, 46, 48, 54). Removing PVAT from the aorta or mesenteric arteries increases the contractile effect of catecholamines on vessel constriction (27, 42). Furthermore, pharmacological denervation mitigates PVAT anticontractile effects induced by EFS (27, 42, 77, 84). The modulation of adipocyte function by norepinephrine, a sympathetic neurotransmitter, supports the concept of functional innervation (35, 42). Conclusions regarding functionality of innervation, however, were reached using indirect methods that targeted PVAT rather than focusing specifically on the direct connection between innervating nerve fibers and adipocytes themselves, while precluding that norepinephrine is derived from neuronal and not nonneuronal sources within the tissue.

Sympathetic activation is implicated in lipolysis with norepinephrine released acting through β-adrenergic receptors to stimulate lipolysis (65–67, 78, 85–91). Interruption of this sympathetic input to adipocytes results in reduced lipolysis and increased adipose tissue size and cellularity, and risk of obesity (65, 67, 78, 85, 86). Disruption of sympathetic signaling to adipose tissue results in decreased lipolysis and increased adiposity (86, 92–94). Clinically, dysregulation or dysfunction of sympathetic signaling to peripheral organ systems is observed in patients suffering from spinal cord injury. Contusions to the spinal cord can result in partial or full severing of autonomic innervation to peripheral organs including adipose tissue. Remodeling of the enteric nervous system occurs following spinal cord injury (95). Survivors of spinal cord injury also have an increased risk of neurogenic metabolic disorders and obesity, as well as potentially suffering from chronic inflammation (96–99). Visceral and subcutaneous adipose tissue size increases in survivors, with the level of plasma proinflammatory adipokines and cardiometabolic markers significantly correlating with injury level (100). Obesity is known to contribute to chronic systemic inflammation (101). That spinal cord injury increases the risk of developing metabolic disorders, obesity, and chronic systemic inflammation, of which adipocytes play a key role, suggests that there exists functional sympathetic innervation to adipose tissue. However, direct evidence remains lacking, while discounting the possibility of other contributing mechanisms.

In the present study, we examined functional neural regulation of adipocyte responses by measuring adipocyte calcium responses following EFS-induced nerve depolarization. Our findings demonstrate that EFS of ex vivo aPVAT, mPVAT, and WAT does not induce robust calcium responses in adipocytes themselves. This limited neuroregulation of adipocytes suggests that functional connectivity between neurons and adipocytes is lacking. Combined, our molecular, histological, and functional analyses suggest that PVAT and WAT do not meet the established criterion to be considered classically innervated.

This study’s findings challenge prevailing beliefs regarding the role of sympathetic innervation in modulating adipocyte function and driving its anticontractile effect in PVAT. The primary evidence supporting the sympathetic-driven anticontractile effects of PVAT comes from studies demonstrating the loss of these effects with sympathetic denervation (42, 55, 69, 71). This simultaneous loss of sympathetic input coupled with PVAT anticontractile function suggests that the connector between the upstream input, electrical stimulation, and downstream output, adipocyte anticontractile function, is sympathetic innervation. However, evidence of direct connections between sympathetic nerves and adipocytes in PVAT remains lacking.

Notably, electrical field stimulation depolarizes all nerve fibers present, not solely sympathetic nerve fibers. Although sympathetic nerves are thought to comprise the majority of innervation present in PVAT, and sympathetic denervation studies argue for their predominating role in driving anticontractile effects, other types of nerve fibers are present that could exert effects on adipocytes alongside sympathetic fibers (44, 45, 48, 66). Interestingly, not all electrical stimulation is equal in exerting the expected anticontractile effects as stronger and longer electrical stimulation of the mesenteric artery triggers a procontractile effect on the vasculature as opposed to the expected anticontractile effect generated with shorter, weaker stimulation (69, 71).

Although this duality in response to electrical stimulation is suggested to indicate that greater neural activity, specifically sympathetic activation, actively alters PVAT function rather than solely driving a specific response, this conclusion hinges on the assumption of two conventional dogmas. The first, that a large degree of innervation and connectivity exists between nerves and adipocytes and the second, that this innervation and connectivity is associated with a corresponding widespread effect of neural activation on adipocyte function. Both assumptions discount existing evidence of limited neural innervation in PVAT, as well as the direct effects these nerve fibers can have on the vasculature and other cell types present in the adipose tissue.

Our anatomical studies support the concept of limited innervation of adipocytes (70) and calcium imaging studies indicate that electrical field stimulation of adipose tissue fails to yield substantial adipocyte responses, aligning with the anatomical data. In addition, confirmation that electrical field stimulation of mesenteric PVAT activates nerve fibers, as evidence by increased calcium responses within those fibers, and triggers mesenteric artery contraction, lends further credence that adipocytes are not directly functionally regulated by nerve fibers present with the tissue.

Although EFS-induced contraction of the mesenteric artery is small in this study, this is likely due to the preparation of the tissue for vascular imaging, with one small piece of PVAT removed above the vessel to better visualize the tissue, while leaving the remaining PVAT intact, thus yielding a mixed effect of vessel contraction due to the electrical current and known anticontractile effect of intact PVAT on vessel contraction. Thus, our combined data argue the limited neural innervation present outside the direct periphery of the vasculature likely plays a minimal role in directly modulating adipocyte function or, alternately, that neuromodulation of adipocytes involves signaling mechanisms independent of those requiring calcium.

Results generated from this study also highlight the importance of heterogeneity between adipose depots. PVAT exhibits unique phenotypes depending on its anatomical location because of differences in adipocyte progenitors, differentiation, and cellular morphology and composition. Composed primarily of brown adipocytes, aPVAT resembles more brown-like adipose tissue, whereas mPVAT is comprised primarily of white adipocytes and resembles more white-like adipose tissue (10, 11). These regional differences could affect gene expression in and responses of adipocytes to stimuli, as well as the mechanisms involved in regulation function. Differences in the expression of genes encoding neurotransmission were detected between all three regions of adipose tissue, male and female, examined, including a higher basal level of expression of α_1a_-adrenergic receptor in aPVAT and lower level of expression in WAT, with mPVAT expression falling in between. Regional differences in adipocyte calcium responses to norepinephrine, bethanechol, and EFS were also observed in both males and females. Although not numerous, some sex differences between males and females were also seen in both gene expression and calcium responses. These data combined underscore the importance that biological sex and anatomical region specialization may play in adipocyte function, establishing a foundation for the development of regional and sex-specific clinical interventions.

Limitations in experimental design do exist in this study that should be weighed when considering the stated results and conclusions drawn. Regarding the molecular analyses run, whole adipose tissue was taken for RNA extraction and gene expression assays. As such, the detection of expressed genes must be considered within the context of the entire tissue, as opposed to specific cells within the tissue. Many of the genes identified in this study are associated with neurotransmission; however, not exclusively. These genes are also expressed in nonneuronal cells and therefore function in roles outside of neurotransmission. A subset of genes of interest were selected for analysis from a RNAseq database and therefore do not comprise an exhaustive examination of the expression of all neurotransmission-associated genes present in PVAT. Although we detected the expression of genes associated with encoding synaptic transmission within PVAT and WAT, we cannot make any definitive conclusions regarding the ability of adipocytes, themselves, to express elements that would enable synaptic communication with nearby neurites. In addition, while changes in basal expression of genes were found, no definitive patterns regarding variations of specific classifications of genes between tissue could be detected, most likely because of the contribution of all cells found within adipose tissue. To better determine if adipocytes can function as postsynaptic cells and if true regional differences exist in synaptic components, molecular analyses should be conducted using adipocytes isolated from whole tissue. Immunofluorescence studies carried out were performed using a pan-neuronal marker on a larger scale, thereby preventing the detection of smaller synaptic structures. Confirmational studies using more appropriate imaging techniques to visualize synapses should be conducted to show the presence or absence of neural synapses within adipose tissue. Conversely, peripheral neurons can regulate cell function in the absence of establishing direct synapses with cells (102). Thus, a lack of classical synaptic structure does not eliminate the potential for peripheral nerve regulation of adipocytes. Importantly, antibodies and transgenic mice used within this study may not label all peripheral nerve fibers including those in adipose tissue because of differences in expression and lineage (61, 103–105), providing a potentially limited and incomplete anatomical map of adipose tissue innervation. Additional studies using additional antibodies, transgenic animals, and adenoviral tracing of peripheral nerves should be conducted to confirm study findings. This study was conducted using healthy mice aged 8–13 wk from a C57Bl6J background, therefore factors such as strain, development, aging, and disease state are not considered here, but will be included in future studies. More nuanced sex-dependent variables such as reproductive history and the estrus cycle were also not examined in this study. The estrus cycle influences white adipose distribution, adipocyte hyperplasia, vascular stiffness and tone (106–114). Furthermore, pregnancy is also known to modulate PVAT regulation of vascular tone (32–34, 115–118). Although the female mice included in this study were not subjected to mating, tracking of their estrus cycle was not performed. Although, significant intrafemale differences in this study were not observed and male-female sex differences were limited, whether this was due to female mice being in similar stages of estrus at the time of experiments is not known and could be of particular importance. Therefore, with the absence of this data, the effects of the estrus cycle on experimental outcomes remain unknown and should be included in future studies. Finally, neural innervation in the vasculature can potentially modulate the flow of nutrients to and from the surrounding tissue. This alteration of nutrient flow in PVAT could influence adipocyte function. Although our findings suggest that adipocytes are not directly functionally regulated by innervation present in PVAT, we have not eliminated the possibility that adipocyte function could be affected by changes in nutrient flow within PVAT in response to neuromodulation of the vasculature. Furthermore, the mechanisms by which norepinephrine exert its effects on adipocytes were not examined in this current study. Functional connectivity between neurons and adipocytes was assessed by visualizing changes in adipocyte intracellular calcium flux. However, neuromodulation of adipocyte function may occur through calcium-independent mechanisms. Future studies examining the role of calcium-independent mechanisms in neuromodulation of adipocyte function will be conducted. In addition, studies incorporating α- and β-receptor antagonists to determine the role that various α- and β-receptors play in norepinephrine stimulated calcium responses in adipocytes are currently underway.

As this study focused on the potential for neural innervation serving as the source of the norepinephrine driving adipocyte anticontractile effects, alternative sources of norepinephrine were not examined. However, this study’s findings suggest that norepinephrine is not likely of neural origin, highlighting the importance of these alternative sources including nonneuronal tissue resident cells. When stimulated, these cells could potentially act in a paracrine fashion, releasing norepinephrine produced and stored within the cells, triggering adipocyte release of anticontractile factors. Furthermore, the source of norepinephrine could come from the adipocytes themselves, releasing intracellular norepinephrine stores that could then activate the releasing adipocyte and neighboring adipocytes to release anticontractile factors. Lending credence to this hypothesis are studies demonstrating that mesenteric adipocytes express enzymes involved in norepinephrine synthesis (85, 119–124). Although an initial attempt to visualize norepinephrine-containing cells in adipose tissue using immunofluorescence in this study failed, efforts to identify norepinephrine producing and releasing cells within perivascular adipose tissue and to define their mechanistic relationship with adipocytes and adipocyte anticontractile effects on the vasculature is currently underway.

Overall, this study demonstrates that with the limited innervation of adipose tissue and lack of direct functional neural regulation of adipocytes, PVAT is not classically innervated, emphasizing the importance that alternative mechanisms may be playing in modulating adipocyte function.

## DATA AVAILABILITY

Source data for this study are available upon request from the corresponding author.

## GRANTS

Research reported in this publication was supported by National Institutes of Health (NIH) Grants P01HL15291 and T32ES007255.

## DISCLAIMERS

The content is solely the responsibility of the authors and does not necessarily represent the official views of the National Institutes of Health.

## DISCLOSURES

No conflicts of interest, financial or otherwise, are declared by the authors.

## AUTHOR CONTRIBUTIONS

S.W.W., W.F.J., and B.D.G. conceived and designed research; M.H., W.M.-S., W.F.J., and B.D.G. performed experiments; M.H., W.M.-S., W.F.J., and B.D.G. analyzed data; M.H. interpreted results of experiments; M.H. prepared figures; M.H. drafted manuscript; M.H., W.M.-S., S.W.W., W.F.J., and B.D.G. edited and revised manuscript; M.H., W.M.-S., S.W.W., W.F.J., and B.D.G. approved final version of manuscript.
